# Supplementary material for: Red light-driven electron sacrificial agents-free photoreduction of inert aryl halides via triplet-triplet annihilation
Source: Nat Commun. 2023 Feb 27;14:1102. doi: 10.1038/s41467-023-36679-7 (PMC9968713; doi:10.1038/s41467-023-36679-7)
Supplement: Supplementary file 1 — Supplementary Information [file 41467_2023_36679_MOESM1_ESM.docx]

Supplementary Information

Red Light-Driven Sacrificial Agents-Free Photoreduction of Inert Aryl Halides via Triplet-Triplet Annihilation

Le Zeng^1,2 #^, Ling Huang^1,2 #^, Wenhai Lin^1^, Lin-Han Jiang^2^ and Gang Han^1*^

^1^ Department of Biochemistry and Molecular Pharmacology, University of Massachusetts Chan Medical School, Worcester, MA 01605, United States.

^2^ Research Center for Analytical Sciences and Tianjin Key Laboratory of Biosensing and Molecular Recognition, College of Chemistry, Nankai University; Haihe Laboratory of Sustainable Chemical Transformations, Tianjin 300192, China.

Contents

[Supplementary Methods 2](#_Toc124958803)

[Supplementary Tables 20](#_Toc124958804)

[Supplementary Figures 25](#_Toc124958805)

[Supplementary References 68](#_Toc124958806)

# Supplementary Methods

**Chemicals:** Perylene, N-bromosuccinimide (NBS), cuprous iodide (CuI), dichloroditriphenyl phosphine palladium (Pd(PPh_3_)_2_Cl_2_), phenylboronic acid, 2-methylbenzene boric acid, potassium carbonate (K_2_CO_3_), sodium hydroxide (NaOH), potassium hydroxide (KOH), cesium carbonate (CsCO_3_), lithium hydroxide (LiOH), sodium bicarbonate (NaHCO_3_), triphenylphosphine (PPh_3_), 4-bromoacetophenone, 4-bromobenzaldehyde, 4-bromobenzonitrile, 2-bromophenonitrile, 2-bromothiophenonitrile, 4-chlorophenonitrile, 4-chlorophenone, 2, 4-dimethylpyrrole, *N*-methylpyrrole, *N*-phenylpyrrole, N-boc-pyrrole, 1, 3, 5-trimethoxybenzene, 2-methylindole, triethoxyl phosphine, toluene, dichloromethane (DCM), tetrahydrofuran (THF), ethanol (EtOH), *N, N*-dimethylformamide (DMF), dimethyl sulfoxide (DMSO) were purchased from Sigma-Aldrich (St. Louis, MO, USA). Ultrapure water was prepared by using a Millipore Simplicity System (Millipore, Bedford, USA). All the above-mentioned chemicals were used as received without further purification.

**Characterization:** ^1^H NMR spectra were recorded with a Bruker 500 MHz spectrometer. UV-vis spectra were recorded on an Agilent Cary-5 spectrophotometer. Steady-state fluorescence spectra were measured on a Hitachi F-7000 fluorescence spectrometer (Xe lamp 500 W). The fluorescence lifetime of the Py0-Py4 was tested on an FLS-1000 spectrometer instrument in Edinburgh coupled to a 405 nm picosecond laser. Triplet-triplet annihilation upconversion spectra were measured on an Edinburgh FLS-1000 fluorescence spectrometer, which is connected to externally 653 nm diode laser as the excitation source (Changchun New Industries Optoelectronics Technology Co., Ltd. China). Upconversion delayed fluorescence lifetimes were characterized on an Edinburgh FLS-1000 spectrometer by coupling a nanosecond laser at 653 nm. 656 nm red LED (Mightex LED, USA) was also used to test the photoactivation experiments of inert aryl halides.

The preparation process of Py1-Py4. (1) 2 equivalent phenylboronic acid, K_2_CO_3_, Pd (PPh_3_)_4_; (2) 2 equivalent 2-methylphenylboronic acid, K_2_CO_3_, Pd (PPh_3_)_4_; (3) 4 equivalent phenylboronic acid, K_2_CO_3_, Pd (PPh_3_)_4_; (4) 4 equivalent 2-methylphenylboronic acid, K_2_CO_3_, Pd (PPh_3_)_4_.

**General synthesis procedure of Py1-Py4**^1^

Under Ar atmosphere, 1-bromoperylene (0.5 mmol, 330 mg) and phenylboronic acid (1 mmol, 122 mg) were dissolved in toluene/ethanol/water (10 mL/10 mL/5 mL). Then K_2_CO_3_(1.5 mmol, 210 mg) and Pd (PPh_3_)_4_ (0.030 mmol, 30 mg) were added. The reaction mixture was refluxed for 12 h. After cooling to room temperature (rt), most of the solvent were evaporated under reduced pressure. Water (100 mL) was added to the residue and the product was extracted using CH_2_Cl_2_ (3×100 mL). The organic phase was dried over Na_2_SO_4_ and then the solvent was evaporated under reduced pressure. The crude products thus obtained were purified by column chromatography (silica gel, CH_2_Cl_2_/n-hexane, 1/3, v/v).

**Py1**: ^1^H NMR (CDCl_3_, 500 MHz) δ = 8.25-8.21 (m, 4H), 7.77-7.76 (d, *J* = 5.0 Hz, 1H), 7.70-7.69 (d, *J* = 5.0 Hz, 2H), 7.53-7.41 ppm (m, 9H); ^13^C NMR (CDCl_3_, 125 MHz) δ = 140.8, 140.0, 134.7, 132.9, 131.4, 131.3, 130.7, 130.0, 129.1, 128.7, 128.4, 127.8, 127.7, 127.4, 126.6, 126.5, 126.1, 120.3, 120.1, 119.9 ppm. ESI-HRMS: [C_26_H_16_ -e] ^+^, calculation: 328.1247, found: 328.1247.

**Py2**: ^1^H NMR (CDCl_3_, 500 MHz) δ = 8.29-8.22 (m, 4H), 7.79-7.75 (m, 2H), 7.55-7.43 ppm (m, 14H); ^13^C NMR (CDCl_3_, 125 MHz) δ = 140.8, 139.9, 132.9, 131.6, 131.4, 130.7, 130.0, 128.9, 128.4, 127.8, 127.4, 126.6, 126.0, 120.5, 120.3, 120.1, 119.9 ppm. ESI-HRMS: [C_32_H_20_ -e] ^+^, calculation: 404.1565, found: 404.1550.

**Py3**: ^1^H NMR (CDCl_3_, 500 MHz) δ = 8.25-8.18 (m, 4H), 7.70-7.69 (d, *J* = 5.0 Hz, 2H), 7.52-7.46 (m, 2H), 7.37-7.27 (m, 7H), 2.09 ppm (s, 3H); ^13^C NMR (CDCl_3_, 125 MHz) δ = 140.2, 139.6, 136.8, 134.8, 133.3, 131.4, 131.3, 130.6, 130.3, 130.0, 128.8, 128.7, 127.7, 127.5, 126.6, 126.2, 125.8, 120.3, 120.2, 120.1, 119.9, 20.7 ppm. ESI-HRMS: [C_27_H_18_ -e] ^+^, calculation: 342.1403, found: 342.1404.

**Py4**: ^1^H NMR (CDCl_3_, 500 MHz) δ = 8.28-8.20 (m, 4H), 7.41-7.28 (m, 14H), 2.09 ppm (s, 6H); ^13^C NMR (CDCl_3_, 125 MHz) δ = 140.2, 139.6, 139.5, 136.9, 133.3, 131.6, 131.5, 130.7, 130.6, 130.3, 130.0, 128.7, 127.7, 127.5, 126.6, 126.1, 126.0, 125.8, 125.7, 120.4, 120.1, 120.0, 119.7, 20.1, 20.0 ppm. ESI-HRMS: [C_34_H_24_ -e] ^+^, calculation: 432.1873, found: 432.1873.

**Fluorescence quantum yield for annihilators (Py0-Py4)**: The fluorescence quantum yield of annihilator in toluene was measured according to the well-established method. Quantum yield measurements and calculations were done using Py0 (λ_ex_ = 410 nm, hexane) as the standard chromophores with quantum yield of 0.98. All absorbance of annihilators was set to below 0.1 at 410 nm to avoid self-quenching. Following equation 1 was used for calculation.

$\Phi_{f}= \Phi_{R}\times(\frac{I}{I_{R}})\times(\frac{A_{R}}{A})\times(\frac{n^{2}}{n_{R}^{2}})$………………………………………….……….(1)

where Φ_R_ stands for fluorescence quantum yield of reference, I and I_R_ represent integrated area of emission spectrum at specific lex for sample and for standard respectively, A and A_R_ represent absorbance of the λ_ex_ for sample and standard respectively, n and n_R_ refer to the refractive indices for the solvent of sample and standard respectively. Refractive index values were taken as 1.4969 for toluene, 1.375 for hexane.

**Electrochemical measurements of annihilators (Py0-Py4).**^2^

Cyclic voltammetry experiments were carried out with an electrochemical luminescence analyzer (Tianjin Lanlike chemical & electronics high tech Co. LTD) using a one compartment electrolysis cell containing a glassy carbon working electrode, a platinum wire counter electrode, and a silver-silver nitrate (1.0 M) reference electrode. The experimental mixture contained 1 mM annihilator in CH_2_Cl_2_ (DCM) solution with 0.1 M tetrabutylammonium hexafluorophosphate (n-Bu_4_NPF_6_, TCI chemicals, electrochemical grade) as the supporting electrolyte. Scan rate is 100 mV/s. The redox potential was calibrated against the ferrocenium/ferrocene couple (Fc^+^/Fc, 0.42 V vs SCE in CH_3_CN). The working solution was degassed with argon for 15 min before measurement and then kept under argon gas flow during the measurement.

**Calculation of the standard** **oxidation potential of the annihilators Py0-Py4.**^2^ If the annihilator presented a reversible redox behavior, the standard oxidation (E_ox_) potential was obtained from CV spectrum as the half sum of anodic (E_pa_) and cathodic (E_pc_) peak potentials:

E_ox_ = (E_pa_ + E_pc_) / 2……………………………………………………(2)

If the annihilator exhibited an irreversible redox behavior, the standard oxidation (E_ox_) of annihilator was taken from the peak potential for oxidation event (E_ox_). The oxidation potentials of Py0-Py4 were listed at Supplementary Table 2.

**Evaluation of the standard reduction potential of the annihilators in the excited state.**^2^

The redox potential of the catalyst in its singlet excited state ($E_{\mathrm{ox}}^{*}$(S_1_)) could be estimated from the following equation:

$$E_{\mathrm{OX}}^{*}\left( S_{1} \right)= E_{\mathrm{ox}}^{o}-E_{00}\left( S_{1} \right)\ldots\ldots\ldots\ldots..(3)$$

Where $E_{\mathrm{ox}}^{o}$ is the oxidation potential of catalyst at the ground state, $E_{00}\left( S_{1} \right)$ represent the energy gap between the singlet excited state and the ground state. If $E_{\mathrm{OX}}^{*}$(S_1_) < 0, the photocatalyst is called an excited state reductant. The excited reduction potentials of Py0-Py4 were calculated and listed at Supplementary Table 2.

**Excited state electron transfer analysis by Rehm-Weller** **equation (thermodynamic analysis).**

The electron transfer (ET) between the photocatalyst (Py) and the substrate is the fundamental step in the occurrence of photoreduction of inert aryl halides, and the analysis of this step can be performed using the Rehm-Weller equation.^3^ In our system, the photocatalyst acts as an electron donor (D), and the aryl halide is an electron acceptor (A). The Gibbs free energy (ΔG_et_) was used to assess the ET step between the photocatalyst and the substrate. The singlet (S_1_) and triplet state (T_1_) energy levels of the photocatalyst were utilized to calculate the ΔG_et_.

$\Delta G_{\mathrm{et}}= E_{\mathrm{OX}}\left( D \right)-E_{\mathrm{red}}\left( A \right)-E^{*}$…………………………………………………(4)

Where ΔG_et_ is the Gibbs free energy for the electron transfer from donor to acceptor, E_OX_ (D) and E_red_ (A) are the oxidation potential of the photocatalyst and the reduction potential of the substrate, respectively. E^*^ is the S_1_ or T_1_ state energy level of the photocatalyst. The detailed results are shown in Supplementary Table 2.

**TTA upconversion characterization:** The solution of sensitizer and annihilator was prepared in the glove box and the cuvette was sealed to prevent oxygen-induced TTA-UC quenching. In order to avoid the high concentration of PdTPBP-caused TTA-UC quenching, we fixed the concentration of PdTPBP to 10 μM to optimize the concentration of annihilator. With the increase of the concentration of annihilator, the upconversion intensity increases until saturation is reached, and then the optimal concentration is determined. A continuous diode-pumped solid-state laser (653 nm) was used as the excitation source to couple with FLS-1000 spectrometer for TTA-UC spectra. Additionally, the optimal concentration of annihilators Py0-Py4 were utilized to measure the power-dependent upconversion properties in conjunction with the PdTPBP photosensitizer via neutral filters to adjust the intensity of laser.

**Measurement of the upconversion efficiency (*****η*_UC_) under 653 nm light illumination:** The *η*_UC_ was calculated by using methylene blue (MB) with fluorescence quantum yield of 3% (in methanol) as the standard.^4^ The following equation 5 was utilized,^5^ where *η*_UC_, *A*_unk_, *I*_unk_, and *η*_unk_ represents the upconversion efficiency, the absorbance of sample, the integrated TTA-UC intensity of unknow sample, and refractive index of the solvents:

${}_{\boldsymbol{UC}}\boldsymbol{=}\boldsymbol{2}\boldsymbol{\times}\boldsymbol{\Phi}_{\boldsymbol{f}}\boldsymbol{\times}\left( \frac{\boldsymbol{A}_{\boldsymbol{std}}}{\boldsymbol{A}_{\boldsymbol{unk}}} \right)\boldsymbol{\times}\left( \frac{\boldsymbol{I}_{\boldsymbol{UC}}}{\boldsymbol{I}_{\boldsymbol{std}}} \right)\boldsymbol{\times}\left( \frac{\boldsymbol{\eta}_{\boldsymbol{UC}}}{\boldsymbol{\eta}_{\boldsymbol{std}}} \right)^{\boldsymbol{2}}$……………………………….…….(5)

**The Stern-Volmer quenching experiment**: Mixed solutions containing sensitizer of PdTPBP (10 μM) and different concentrations of annihilator (0, 2.5, 5, 10, 15, 20 μM) were firstly prepared in the glove box. Then the photoluminescence of photosensitizer at 800 nm was measured for these solutions. Equation 6 was used to calculate *k_sv_* constants. *I*_o_ and *I*_t_ stand for photoluminescence intensity of photosensitizer in the absence or presence of annihilators, respectively. Q is the concentration of annihilator. Bimolecular quenching constant (*k_q_*) was calculated by equation 7.^6^ The τ_T_ is the phosphorescence lifetime of photosensitizer in argon. The maximum phosphorescence intensity of PdTPBP is 800 nm, leading to a T_1_ state of 1.55 eV. The intrinsic triplet excited state lifetime of PdTPBP is 223.7 µs.^7^

$\frac{I_{0}}{I_{t}}=1+k_{sv}Q$………………………………………………………………(6)

$k_{sv}= k_{q}\times\tau_{T}$……………………………………………………………..(7)

**Triplet-triplet energy transfer (TTET) efficiency measurements**. The PdTPBP-to-annihilator triplet-triplet energy transfer quantum yield (Φ_TTET_) was calculated using the following equation 8.^8^ *I* and *I_0_* represent the integral phosphorescence of photosensitizer PdTPBP in the presence or absence of the annihilator, respectively.

$\Phi_{\mathrm{TTET}}=1- \frac{I}{I_{0}}$………………………………………………..………………(8)

**The normalized triplet-triplet annihilation quantum yield (*****η*_TTA_) calculation:** *η*_TTA_ was calculated by the following formula.^9^

*η*_UC_ = 2 × *f* × Φ_ISC_ × Φ_TTET_ × Φ_TTA_ × Φ_f_ ………………………………………(9)

In this formula, the *f* is spin statistics factor, Φ_ISC_ is the intersystem crossing quantum yield of the PdTPBP. Φ_TTET_ is triplet-triplet energy transfer quantum yield between PdTPBP and annihilator; Φ_TTA_ is quantum yield of triplet-triplet annihilation for annihilator. Therefore, we can calculate the result of *η*_TTA_ according to formula 9. The Φ_ISC_ for PdTPBP is close to 1, and *η*_UC_, Φ_TTET_ and Φ*_f_* have been calculated according to equation 5, 8, 1.

Therefore,

$\eta_{TTA}=f\times\Phi_{TTA}= \frac{{0.5}_{\mathrm{UC}}}{\Phi_{ISC}\times\Phi_{TTET}\times\Phi_{f}}$…………………………………………(10)

**Kinetic analysis of photoreduction of inert aryl halides via TTA.**

Stern-Volmer quenching constant was calculated via Eq. 6. Using 4-bromoacetophenone as a substrate molecule, the quenching of PdTPBP phosphorescent intensity and TTA-UC intensity were determined in response to various concentration (0, 10, 20, 30, 40, and 50 mM) of the substrate. The bimolecular quenching constant (*k*_q_) between the substrate and the photocatalyst was calculated via Eq. 7 with the fluorescence lifetime change of the photocatalyst (Py).

**Generation and deactivation pathways of ^1^Py^*^**

The absorption ability of PdTPBP (ε _(PdTPBP)_), the intersystem crossing efficiency of PdTPBP (Φ_ISC_), the triplet energy transfer efficiency (Φ_TTET_) between PdTPBP and the photocatalyst (Py), and the triplet-triplet annihilation (Φ_TTA_) between photocatalysts all contribute to the generation of ^1^Py^*^.

^1^Py^*^ ∝ ε _(PdTPBP)_ × Φ_ISC_ × Φ_TTET_ × Φ_TTA_ …………………………………………(11)

Eq. 12 was used to calculate the quantum efficiency of ^1^Py^*^ generation (Φ_1Py*_).

$\Phi_{1\mathrm{Py}*}= \frac{\Phi_{UC}}{\Phi_{f}}$………………………….…………………………………………(12)

Φ_UC_ is the TTA-UC quantum yield (the maximum limit is 50%), and Φ*_f_* is the fluorescence quantum yield of the photocatalyst (Py).

In the presence of 4-bromoacetophenone, the quenching pathway of ^1^Py^*^ is electron transfer between Py and the substrate *k*_q_ (S_1_) × [4-bromoacetophenone], fluorescence radiation of Py (*k*_F_), and non-radiation transition of Py itself (*k*_nr_).^10^

k_D_(S_1_) = *k*_q_ (S_1_) × [4-bromoacetophenone] + *k*_F_ + *k*_nr_ …………………………(13)

The electron transfer between ^1^Py^*^ and 4-bromoacetophenone is primarily responsible for the suppression of TTA-UC emission in the presence of 4-bromoacetophenone. Equation 14 was used to obtain the quantum yield for overall photocatalytic reaction (Φ_overall_)

$\Phi_{\mathrm{overall}}= \Phi_{UC}(without)-\Phi_{UC}$(within)……….……………………………(14)

where Φ_UC_ (without) is the quantum yield of TTA-UC in the absence of 4-bromoacetophenone and Φ_UC_ (within) is the quantum yield of TTA-UC in the presence of 4-bromoacetophenone. Supplementary Table 5 contains the detailed results.

**The activation barrier (Δ*G*^≠^) for the single electron transfer (ET) step from ^1^Py4^*^ to the 4-bromoacetophenone.**

The Δ*G***^≠^** was determined via the Marcus theory.^10^

${\Delta G}^{\neq}= \frac{{(\Delta Get+\boldsymbol{\lambda})}^{2}}{4\boldsymbol{\lambda}}$…………………………………………..…………………(15)

Where, ΔG_et_ is Gibbs free energy of ET between ^1^Py4^*^ and the 4-bromoacetophenone, *λ* is the nuclear reorganization energy and this value is taken from the literature. λ (4-bromoacetophenone) = 24.27 kJ/mol.^10^

ΔG_et_ = -0.06 eV × 96.485 kJ/mol = -5.79 kJ/mol.

Δ*G*^≠^ = (-5.79 + 24.27)^2^ / (4 × 24.27) = 3.52 kJ/mol.

**Back electron transfer process.**

Py4^+●^ → Py4 ΔΕ = + 0.81V

Int b → Int a ΔΕ = − 0.49 V^10^

Py4^+●^ + Int a → Py4+ Int b ΔΕ = + 0.32V.

Therefore: ΔG = –n∙F∙ΔΕ, ΔG < 0, exergonic process.

**Characterization data of the product for the photoactivation of aryl halides**

 Compound **1**: ^1^H NMR (CDCl_3_, 500 MHz) δ = 7.99 (d, *J* = 10.0 Hz, 2H), 7.50 (d, *J* = 10.0 Hz, 2H), 6.76 (s, 1H), 6.35 (s, 1H), 6.23 (s, 1H), 3.71 (s, 3H), 2.61 ppm (s, 3H); ^13^C NMR (CDCl_3_, 125 MHz) δ = 197.7, 138.0, 134.9, 133.4, 128.6, 128.0, 125.4, 110.3, 108.4, 35.5, 26.6 ppm. ESI-HRMS: [C_13_H_13_NO +H]^+^, calculation: 200.1070, found: 200.1060.

Compound **2**: ^1^H NMR (CDCl_3_, 500 MHz) δ = 7.96 (d, *J* = 10.0 Hz, 2H), 7.46 (d, *J* = 10.0 Hz, 2H), 7.38-7.37 (m, 1H), 6.28-6.24 (m, 2H), 2.62 (s, 3H), 1.40 ppm (s, 9H); ^13^C NMR (CDCl_3_, 125 MHz) δ = 197.7, 149.1, 139.0, 135.6, 134.0, 129.1, 127.7, 123.6, 115.6, 110.9, 84.1, 27.7, 26.6 ppm. ESI-HRMS: [C_17_H_19_NO_3_ +H]^+^, calculation: 286.1438, found: 286.1426.

Compound **3**: ^1^H NMR (CDCl_3_, 500 MHz) δ = 7.92 (d, *J* = 10.0 Hz, 2H), 7.42 (d, *J* = 10.0 Hz, 2H), 7.32-7.29 (m, 2H), 7.25-7.24 (m, 1H), 7.01 (d, *J* = 5.0 Hz, 1H), 6.82-6.81 (m, 1H), 6.39-6.38 (m, 1H), 6.31-6.30 (m, 1H), 2.58 ppm (s, 3H); ^13^C NMR (CDCl_3_, 125 MHz) δ = 197.6, 138.4, 137.9, 135.2, 133.8, 128.8, 128.6, 128.2, 127.5, 126.3, 124.7, 110.5, 109.1, 26.6 ppm.

Compound **4**: ^1^H NMR (CDCl_3_, 500 MHz) δ = 7.97 (d, *J* =10.0 Hz, 2H), 7.46 (d, *J* = 10.0 Hz, 2H), 5.87 (s, 1H), 2.60 (s, 3H), 2.31 (s, 3H), 2.28 ppm (s, 3H); ^13^C NMR (CDCl_3_, 125 MHz) δ = 197.4, 138.3,129.3, 129.1, 125.6, 124.7, 122.6, 119.4, 111.4, 26.5, 13.1, 13.0 ppm. ESI-HRMS: [C_14_H_15_NO+H]^+^, calculation: 214.1226, found: 214.1217.

Compound **5**: ^1^H NMR (CDCl_3_, 500 MHz) δ = 7.99 (d, *J* = 10.0 Hz, 2H), 7.45 (d, *J* = 10.0 Hz, 2H), 6.23 (s, 2H), 3.87 (s, 3H), 3.73 (s, 6H), 2.62 ppm (s, 3H); ^13^C NMR (CDCl_3_, 125 MHz) δ = 198.0, 161.1, 158.3, 139.8, 135.1, 131.6, 127.7, 111.3, 90.9, 55.8, 26.6 ppm. ESI-HRMS: [C_17_H_18_O_4_+H]^+^, calculation: 287.1278, found: 287.1267.

Compound **6**: ^1^H NMR (CDCl_3_, 500 MHz) δ = 8.15 (s, 1H), 8.06 (d, *J* = 10.0 Hz, 2H), 7.68 (d, *J* = 10.0 Hz, 2H), 7.63 (d, *J* = 5.0 Hz, 1H), 7.39 (d, *J* = 5.0 Hz, 1H), 7.25-7.23 (m, 1H), 7.16-7.15 (m, 1H), 2.64 (s, 3H), 2.50 ppm (s, 3H); ^13^C NMR (CDCl_3_, 125 MHz) δ = 197.6, 136.2, 135.4, 132.6, 129.9, 128.9, 127.3, 123.2, 119.8, 119.3, 110.9, 110.8, 26.6, 10.0 ppm. ESI-HRMS: [C_17_H_15_NO+H]^+^, calculation: 250.1226, found: 250.1218.

Compound **7**: ^1^H NMR (CDCl_3_, 500 MHz) δ = 8.04-8.02 (m, 2H), 7.94-7.90 (m, 2H), 4.22-4.09 (m, 4H), 2.64 (s, 3H), 1.35-1.32 ppm (m, 6H); ^13^C NMR (CDCl_3_, 125 MHz) δ = 197.5, 139.8, 134.1, 132.1, 132.0, 128.1, 62.4, 26.8, 16.3 ppm. ESI-HRMS: [C_12_H_17_O_4_P+H]^+^, calculation: 257.0937, found: 257.0927.

Compound **8**: ^1^H NMR (CDCl_3_, 500 MHz) δ = 10.01 (s, 1H), 7.90 (d, *J* = 10.0 Hz, 2H), 7.57 (d, *J* = 10.0 Hz, 2H), 6.79-6.78 (m, 1H), 6.39-6.38 (m, 1H), 6.24-6.23 (m, 1H), 3.73 ppm (s, 3H); ^13^C NMR (CDCl_3_, 125 MHz) δ = 191.7, 139.3, 134.3, 133.3, 130.0, 128.2, 125.8, 110.8, 108.6, 35.6 ppm. ESI-HRMS: [C_12_H_11_NO+H]^+^, calculation: 186.0913, found: 186.0905.

Compound **9**: ^1^H NMR (CDCl_3_, 500 MHz) δ = 7.68 (d, *J* = 10.0 Hz, 2H), 7.51 (d, *J* = 10.0 Hz, 2H), 6.78-6.77 (m, 1H), 6.35-6.34 (m, 1H), 6.23-6.22 (m, 1H), 3.71 (s, 3H); ^13^C NMR (CDCl_3_, 125 MHz) δ = 137.7, 132.6, 132.3, 128.3, 125.8, 119.0, 110.8, 109.7, 108.6, 35.5 ppm. ESI-HRMS: [C_12_H_10_N_2_+H]^+^, calculation: 183.0917, found: 183.0910.

Compound **10**: ^1^H NMR (CDCl_3_, 500 MHz) δ = 7.74-7.73 (m, 1H), 7.61-7.59 (m, 1H), 7.44-7.39 (m, 2H), 6.79-6.78 (m, 1H), 6.41-6.40 (m, 1H), 6.25-6.24 (m, 1H), 3.61 ppm (s, 3H); ^13^C NMR (CDCl_3_, 125 MHz) δ = 136.9, 133.5, 132.3, 130.9, 127.4, 124.8, 118.6, 112.9, 111.5, 108.3, 34.8 ppm. ESI-HRMS: [C_12_H_10_N_2_+H]^+^, calculation: 183.0917, found: 183.0908.

Compound **11**: ^1^H NMR (CDCl_3_, 500 MHz) δ = 7.53 (s, 1H), 7.33-7.28 (m, 3H), 7.01 (d, *J* = 5.0 Hz, 2H), 6.85 (d, *J* = 5.0 Hz, 1H), 6.82 (s, 1H), 6.54-6.53 (m, 1H), 6.27-6.26 (m, 1H), 2.50 ppm (s, 3H); ^13^C NMR (CDCl_3_, 125 MHz) δ = 190.4, 143.5, 142.2, 137.8, 133.0, 128.9, 127.6, 126.6, 126.2, 125.6, 124.8, 112.2, 109.3, 26.5 ppm.

Compound **12**: ^1^H NMR (CDCl_3_, 500 MHz) δ = 10.09 (s, 1H), 8.01-7.96 (m, 4H), 4.21-4.10 (m, 4H), 1.38-1.33 ppm (m, 6H); ^13^C NMR (CDCl_3_, 125 MHz) δ = 191.6, 132.4, 132.3, 129.4, 129.3, 62.6, 16.4 ppm. ESI-HRMS: [C_11_H_15_O_4_P+H]^+^, calculation: 243.0781, found: 243.0772.

Compound **13**: ^1^H NMR (CDCl_3_, 500 MHz) δ = 8.14-8.10 (m, 1H), 7.82-7.80 (m, 1H), 7.73-7.61 (m, 2H), 4.28-4.18 (m, 4H), 1.40-1.37 ppm (m, 6H); ^13^C NMR (CDCl_3_, 125 MHz) δ = 135.5, 134.6, 134.4, 132.2, 132.1, 117.1, 114.6, 63.2, 16.3 ppm. ESI-HRMS: [C_11_H_14_NO_3_P+H]^+^, calculation: 240.0784, found: 240.0773.

Compound **14**: ^1^H NMR (CDCl_3_, 500 MHz) δ = 7.95-7.90 (m, 2H), 7.77-7.75 (m, 2H), 4.20-4.08 (m, 4H), 1.35-1.33 ppm (m, 6H); ^13^C NMR (CDCl_3_, 125 MHz) δ = 132.3, 132.2, 132.1, 132.0, 62.7, 16.4 ppm. ESI-HRMS: [C_11_H_14_NO_3_P+H]^+^, calculation: 240.0784, found: 240.0774.

Compound **15**: ^1^H NMR (CDCl_3_, 500 MHz) δ = 7.70-7.69 (m, 1H), 7.65-7.62 (m, 1H), 4.21-4.12 (m, 4H), 2.60 (s, 3H), 1.37-1.34 ppm (m, 6H); ^13^C NMR (CDCl_3_, 125 MHz) δ = 190.6, 150.4, 136.6, 135.2, 132.0, 63.1, 27.2, 16.2 ppm. ESI-HRMS: [C_10_H_15_O_4_PS+H]^+^, calculation: 263.0501, found: 263.0492.

Compound **16**: ^1^H NMR (CDCl_3_, 500 MHz) δ = 9.0 (s, 1H), 8.88 (d, *J* = 5.0 Hz, 1H), 8.38 (s, 1H), 8.30 (d, *J* = 5.0 Hz, 1H), 7.25-7.18 (m, 3H), 7.06 (d, *J* = 10.0 Hz, 1H), 6.80 (s, 1H), 4.42-4.26 (m, 2H), 3.78 (s, 3H), 3.33-3.25 (m, 2H), 3.07-3.00 (m, 4H), 2.66 (s, 1H), 2.61 (s, 1H), 2.48 (s, 3H), 2.32 (s, 3H), 2.28 (s, 3H), 2.14-2.04 (m, 2H), 1.42-1.37 ppm (m, 1H); ^13^C NMR (CDCl_3_, 125 MHz) δ = 165.4, 150.1, 146.7, 135.1, 132.7, 129.8, 129.7, 129.6, 126.3, 125.9, 123.3, 122.2, 121.5, 119.2, 114.9, 111.1, 110.2, 109.0, 73.6, 70.0, 68.1, 53.4, 49.5, 43.7, 41.0, 32.8, 31.4, 30.1, 22.3, 14.2, 12.8 ppm. ESI-HRMS: [C_30_H_34_N_4_O_3_+H]^+^, calculation: 499.2704, found: 499.2688.

Compound **17**: ^1^H NMR (CDCl_3_, 500 MHz) δ = 9.37 (s, 1H), 9.16-9.14 (m, 1H), 8.69 (d, *J* = 10.0 Hz, 1H), 7.25-7.21 (m, 1H), 7.20-7.18 (m, 1H), 7.06 (d, J=10.0 Hz, 1H), 6.80 (s, 1H), 4.42-4.25 (m, 2H), 4.22-4.10 (m, 4H), 3.78 (s, 3H), 3.25-3.19 (m, 2H), 3.06-3.00 (m, 4H), 2.65 (s, 1H), 2.49 (s, 3H), 2.38 (s, 1H), 2.13-2.04 (m, 2H), 1.38-1.35 (m, 7H); ^13^C NMR (CDCl_3_, 125 MHz) δ = 164.3, 155.7, 155.6, 153.7, 140.3, 135.1, 129.6, 126.2, 125.9, 123.3, 121.4, 114.9, 110.2, 109.0, 73.6, 70.0, 68.5, 60.6, 49.5, 43.8, 32.8, 31.5, 30.2, 22.3, 20.9, 16.4, 14.2 ppm. ESI-HRMS: [C_28_H_36_N_3_O_6_P+H]^+^, calculation: 542.2414, found: 542.2400.

# Supplementary Tables

**Supplementary Table 1**. Optimization of the perylene-photocatalyzed photoactivation of aryl halides.

| Solvent | Base | Reaction time (hour) | Yield *^c^* |
| --- | --- | --- | --- |
| DMSO | K_2_CO_3_ | 12 | 51.7 |
| DMSO | K_2_CO_3_ | 20 | 69.5 |
| DMSO *^a^* | K_2_CO_3_ | 20 | 0 |
| DMSO | - | 20 | 6 |
| DMSO *^b^* | K_2_CO_3_ | 20 | 0 |
| DMSO | Na_2_CO_3_ | 20 | 59.4 |
| DMSO | Li_2_CO_3_ | 20 | 57.5 |
| DMSO | Cs_2_CO_3_ | 20 | 46.2 |
| DMSO | NaHCO_3_ | 20 | 58.8 |
| DMSO | NaOH | 20 | 0 |
| DMF | K_2_CO_3_ | 20 | 47.1 |
| DMA | K_2_CO_3_ | 20 | 62.0 |
| Acetone | K_2_CO_3_ | 20 | 27.3 |
| CH_3_CN | K_2_CO_3_ | 20 | 32.8 |
| 1,4-dioxane | K_2_CO_3_ | 20 | 0 |

Reaction condition: 4-bromoacetophenone (0.5 mmol), *N*-methyl pyrrole (5.0 mmol), base (1.0 mmol), solvent (2 mL), 455 nm LED (60 mW/cm^2^), in argon, perylene (5 mM). ^a^ no light; ^b^ no photocatalyst; *^c^* isolated yield.

**Supplementary Table 2**. Free energies for excited-state electron transfer from Py (S_1_) and Py (T_1_) to 4-bromoacetophenone.

| Compound | *E*_OX_ (V) *^a^* | *E*_OX_ (S_1_) (V)*^b^* | *E*_OX_ (T_1_) (V)*^c^* | ΔG_et_ (S_1_) (V) *^d^* | ΔG_et_ (T_1_) (V) *^e^* |
| --- | --- | --- | --- | --- | --- |
| Py0 | +0.90 | -1.88 | -0.63 | -0.04 | +1.21 |
| Py1 | +0.88 | -1.85 | -0.63 | -0.01 | +1.21 |
| Py2 | +0.63 | -2.01 | -0.85 | -0.17 | +0.99 |
| Py3 | +0.90 | -1.85 | -0.63 | -0.01 | +1.21 |
| Py4 | +0.81 | -1.90 | -0.71 | -0.06 | +1.13 |

*^a^* Oxidation potential of Py0-Py4 (*vs.* SCE). *^b^* Oxidation potential of Py0-Py4 in the singlet excited state (S_1_). The energy level of S_1_ is estimated using the intersection of absorption and emission spectra. *^c^* Oxidation potential of Py0-Py4 in the triplet excited state (T_1_). T_1_ energy level is estimated using TD-DFT calculation. *^d^* The Gibbs free energy of electron transfer between the S_1_ state of the photocatalyst and 4-bromoacetophenone. *E* _red_ (4-bromoacetophenone) = −1.84 V vs SCE. *^e^* The Gibbs free energy of electron transfer between the T_1_ state of the photocatalyst and 4-bromoacetophenone.

**Supplementary Table 3**. Photophysical properties of PdTPBP and Py0-Py4 in toluene *^a^*.

|  | *λ*_abs_ *^b^* | *ε* *^c^* | *λ*_em_ *^d^* | Φ*_f_* *^e^* | Φ*_f_* *^f^* | τ*_f_* *^g^* | S_1_^cal^ *^h^* | S_1_^exp^ *^i^* | T_1_^cal^ *^j^* | 2×T_1_–S_1_ (eV) |
| --- | --- | --- | --- | --- | --- | --- | --- | --- | --- | --- |
| PdTPBP | 628 | 1.1 | 795 | _ |  | _ | _ | _ | 1.56 | _ |
| Py0 | 439 | 0.37 | 451 | 83 | 82 | 3.89 | 2.87 | 2.78 | 1.53 | 0.28 |
| Py1 | 447 | 0.35 | 467 | 88 | 87 | 3.37 | 2.70 | 2.73 | 1.51 | 0.29 |
| Py2 | 455 | 0.34 | 484 | 71 | 72 | 3.27 | 2.60 | 2.64 | 1.48 | 0.28 |
| Py3 | 445 | 0.28 | 460 | 84 | 83 | 3.56 | 2.85 | 2.75 | 1.53 | 0.31 |
| Py4 | 451 | 0.25 | 471 | 75 | 76 | 3.08 | 2.70 | 2.71 | 1.52 | 0.34 |

*^a^* in toluene; *^b^* maximum absorption wavelength (nm); *^c^* molar extinction coefficient (10^5^, M^–1^ cm^–1^); *^d^* maximum fluorescence emission wavelength (nm); *^e^* fluorescence quantum yield in toluene (%); *^f^* fluorescence quantum yield in DMSO (%); *^g^* fluorescence lifetime (ns); *^h^* singlet excited state (S_1_) was calculated by TD-DFT in toluene (eV); *^i^* S_1_ energy level was calculated using the intersection of fluorescence emission and UV-vis absorption spectra (eV); *^j^* the lowest-lying triplet state energy (T_1_) was calculated by TD-DFT in toluene (eV).

**Supplementary Table 4**. DFT-Calculation for the Py1-Py4. The electronic excitation energies (eV) and corresponding oscillator strengths (*f*), main configurations, and CI coefficients of the low-lying electronic excited states are given. *^a^*

|  |  | Electronic transition *^b^* | Energy *^c^* | *f ^d^* | CI *^e^* | Composition *^f^* |
| --- | --- | --- | --- | --- | --- | --- |
| Py1 | Singlet | S_o_ → S_1_ | 2.70 eV / 459.63 nm | 0.6692 | 0.70614 | H → L |
|  |  | S_o_ → S_2_ | 3.67 eV / 338.10 nm | 0.0001 | 0.47228 | H → L+1 |
|  | Triplet | T_o_ → T_1_ | 1.51 eV/ 819.59 nm | 0.0000*^g^* | 0.69691 | H → L |
|  |  | T_o_ → T_2_ | 2.98 eV / 415.70 nm | 0.0000*^g^* | 0.37717 | H−1 → L |
| Py2 | Singlet | S_o_ → S_1_ | 2.60 eV/ 476.18 nm | 0.8595 | 0.70599 | H → L |
|  |  | S_o_ → S_2_ | 3.62 eV / 342.54 nm | 0.0000 | 0.60591 | H → L+2 |
|  | Triplet | T_o_ → T_1_ | 1.48 eV / 837.50 nm | 0.0000*^g^* | 0.69609 | H → L |
|  |  | T_o_ → T_2_ | 2.95 eV / 420.38 nm | 0.0000*^g^* | 0.50530 | H → L+1 |
| Py3 | Singlet | S_o_ → S_1_ | 2.75 eV / 451.37 nm | 0.6193 | 0.70612 | H → L |
|  |  | S_o_ → S_2_ | 3.69 eV / 336.08 nm | 0.0001 | 0.58426 | H → L+1 |
|  | Triplet | T_o_ → T_1_ | 1.53 eV / 809.65 nm | 0.0000*^g^* | 0.69792 | H → L |
|  |  | T_o_ → T_2_ | 3.06 eV / 405.68 nm | 0.0000*^g^* | 0.41887 | H → L+2 |
| Py4 | Singlet | S_o_ → S_1_ | 2.70 eV/ 459.55 nm | 0.7555 | 0.70586 | H → L |
|  |  | S_o_ → S_2_ | 3.67 eV / 338.19 nm | 0.0000 | 0.58611 | H → L+1 |
|  | Triplet | T_o_ → T_1_ | 1.52 eV / 816.48 nm | 0.0000*^g^* | 0.69755 | H → L |
|  |  | T_o_ → T_2_ | 3.03 eV / 408.67 nm | 0.0000*^g^* | 0.41228 | H → L+2 |

*^a^* The electronic transitions were calculated by TD-DFT//B3LYP/6-31G(d), based on the DFT//B3LYP/6-31G(d)-optimized ground state and excited geometries. *^b^* TD-DFT//B3LYP/6-31G(d), based on the DFT//B3LYP/6-31G(d)-optimized ground state geometries. *^c^* Only the selected low-lying excited states are presented. *^d^* Oscillator strengths. *^e^* CI coefficients are in absolute values. *^f^* TD-DFT//B3LYP/6-31G(d)-optimized excited state geometries. *^g^* No spin-orbital coupling effect was considered; thus, the *f* values are zero.

**Supplementary Table 5**. The quantum yield for the overall photocatalytic reaction with PdTPBP/Py (Py0-Py4) as the photocatalyst pairs in DMSO.

| **An** | Φ_Py*_ (%) *^a^* | Φ_UC (without)_ (%) *^b^* | Φ_UC (within)_ (%) *^c^* | Φ_overall_ (%) *^d^* | contribution (%) *^e^* |
| --- | --- | --- | --- | --- | --- |
| **Py0** | 3.5 | 2.9 | 2.3 | 0.6 | 20.7 |
| **Py1** | 5.3 | 4.6 | 3.7 | 0.9 | 19.6 |
| **Py2** | 11.0 | 7.9 | 6.3 | 1.6 | 20.2 |
| **Py3** | 8.9 | 7.4 | 5.9 | 1.5 | 20.2 |
| **Py4** | 12.8 | 9.7 | 7.8 | 1.9 | 19.6 |

*^a^* Quantum efficiency of the generation of singlet excited Py (Py^*^) via TTA mechanism; *^b^* TTA-UC quantum yield in the absence of 4-bromoacetophenone. PdTPBP: 10 μM, Py: 500 μM. *^c^* TTA-UC quantum yield in the presence of 4-bromoacetophenone (50 mM). PdTPBP: 10 μM, Py: 500 μM. *^d^* The quantum yield for the overall photocatalytic reaction of Py0-Py4 in conjugation with PdTPBP. *^e^* The percentage of TTA-generated singlet excited Py for the activation of inert aryl halide.

# Supplementary Figures


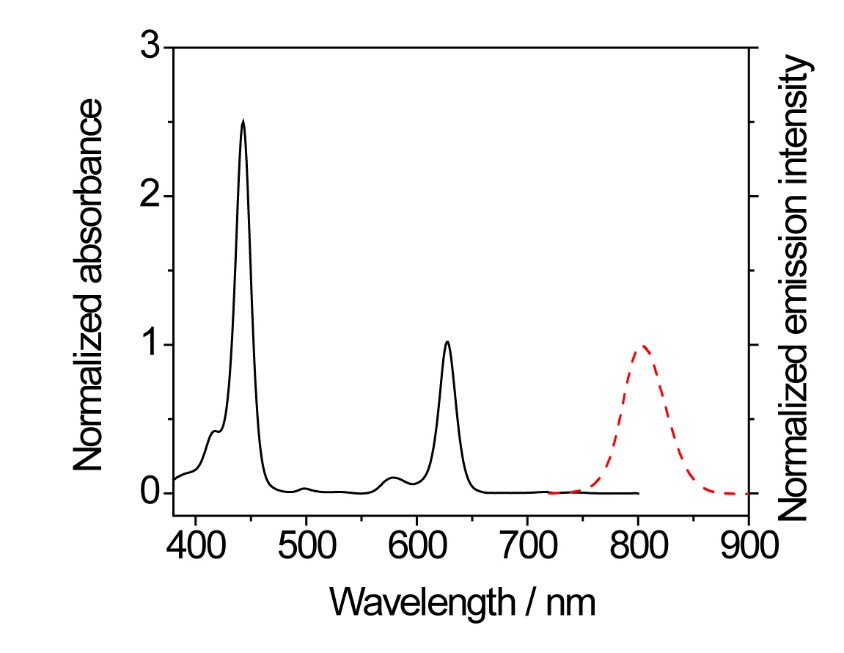


**Supplementary Figure 1**. Normalized UV-vis absorption and emission spectra of PdTPBP in toluene, *λ*_ex_ = 630 nm.


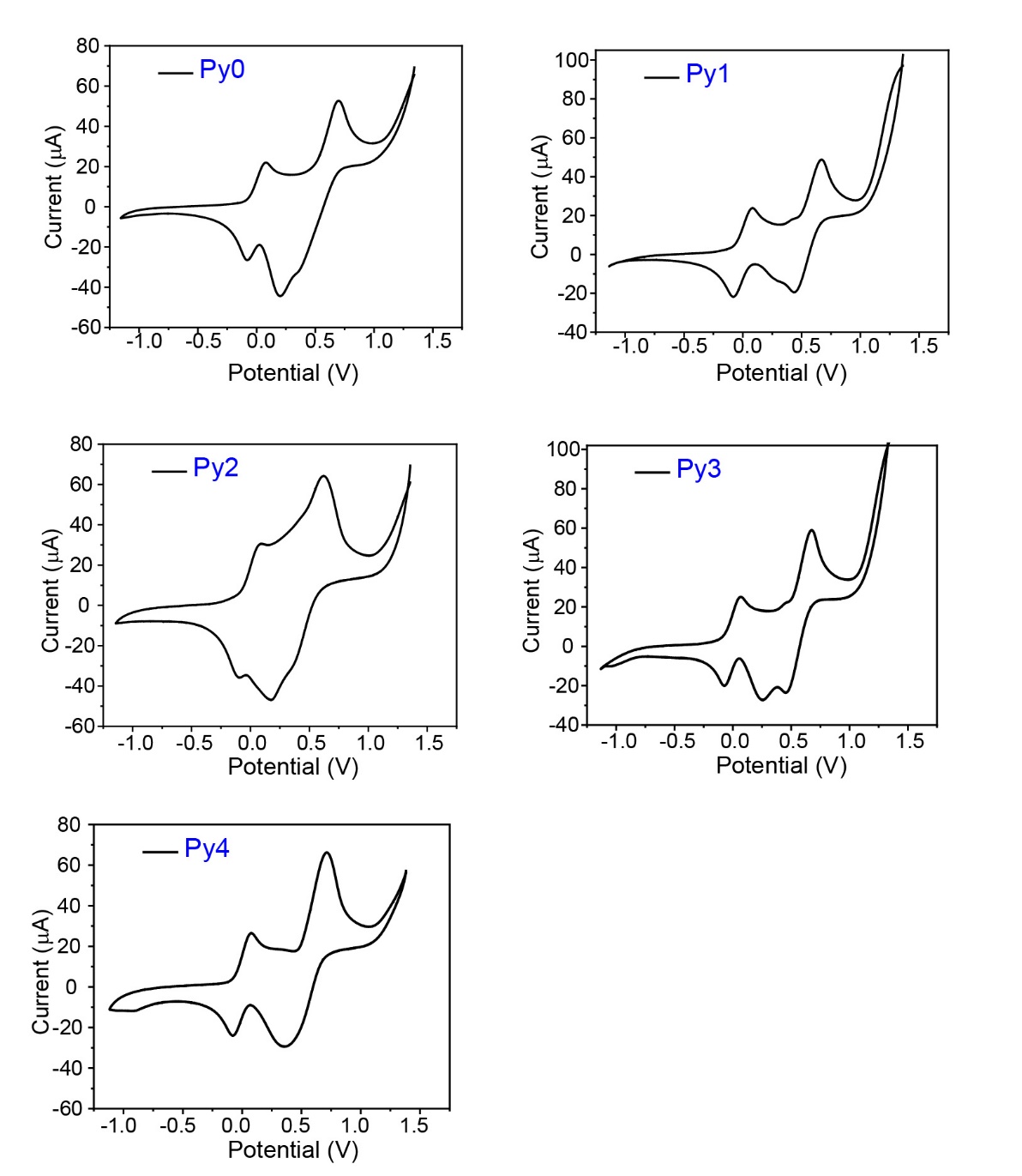


**Supplementary Figure 2**. Cyclic voltammogram of Py0-Py4 in DCM at RT.


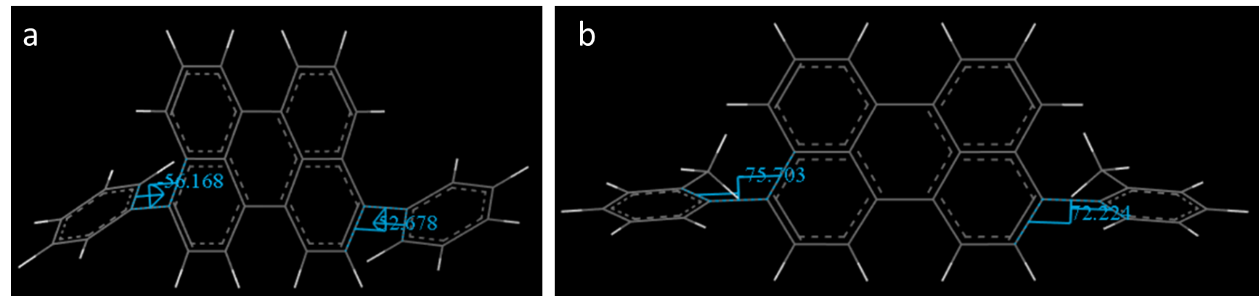


**Supplementary Figure 3**. Optimized conformations of (a)Py2 and (b) Py4 calculated by DFT at the B3LYP/6-31G (d) level with Gaussian 09.


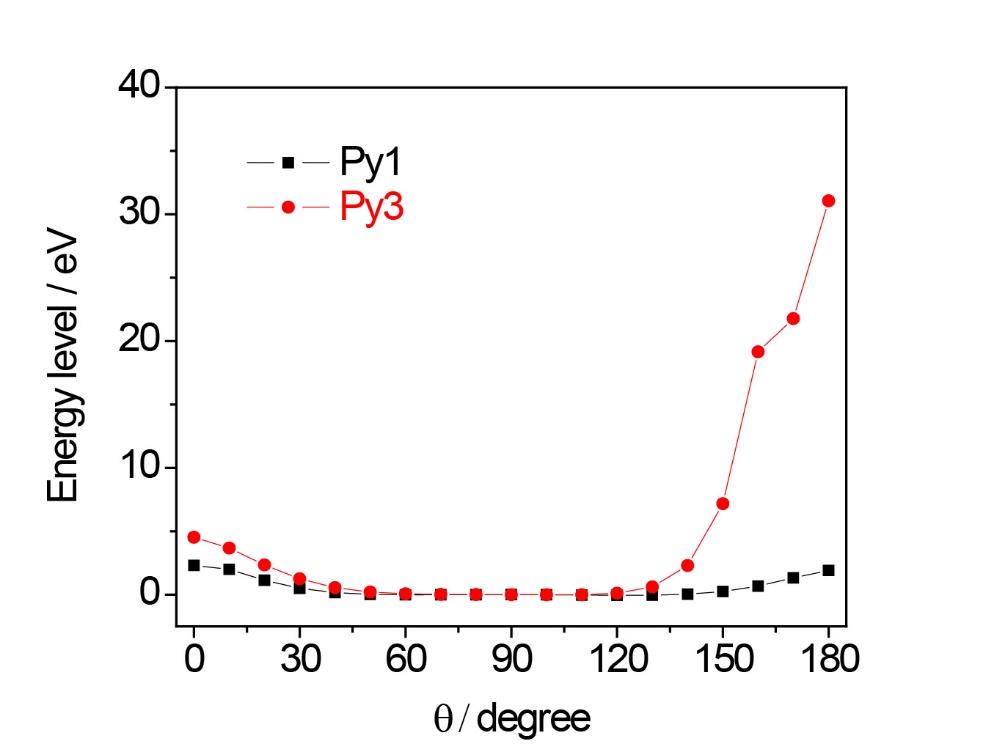


**Supplementary Figure 4**. B3LYP/6-31G(d) calculated potential energy curves of Py1 and Py3 at ground state. θ is the dihedral angle between perylene and phenyl moiety.


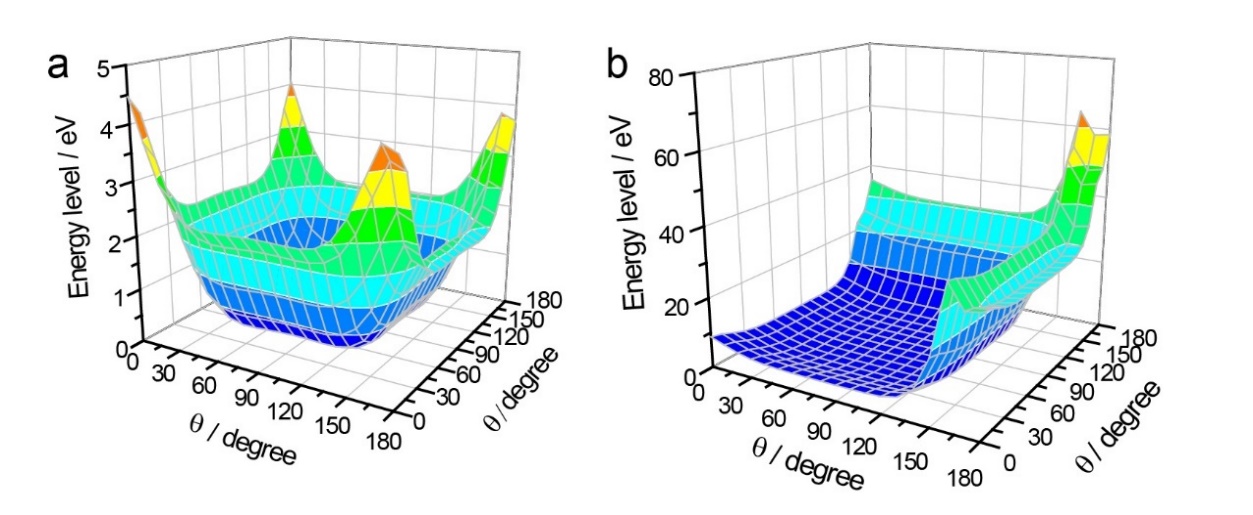


**Supplementary Figure 5**. B3LYP/6-31G(d) calculated potential energy curves of (a) Py2 and (b) Py4 at ground state. θ is the dihedral angle between perylene and phenyl moiety.


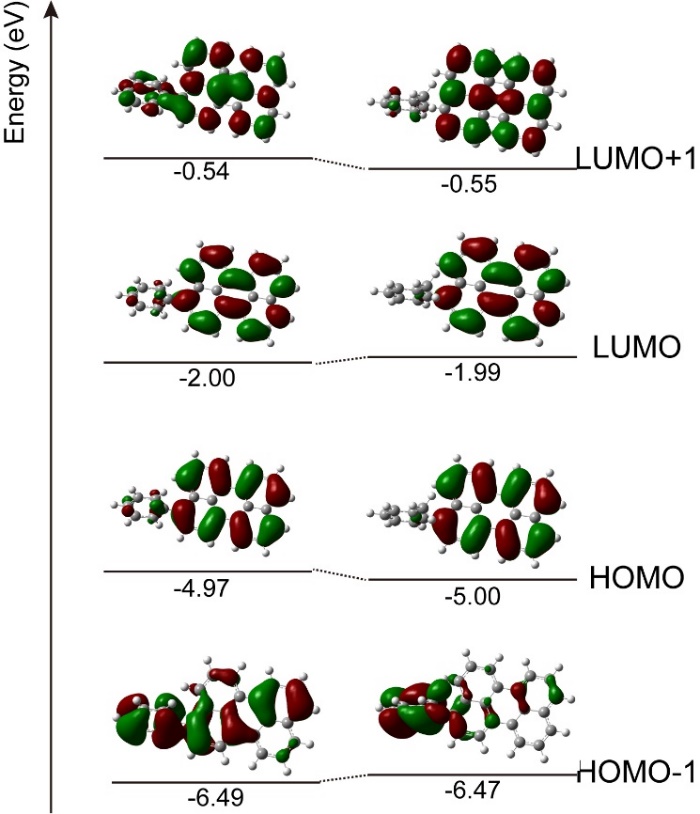


**Supplementary Figure 6**. Frontier molecular orbitals of Py1(left) and Py3 (right) on the basis of the optimized ground-state geometry at the B3LYP/6-31G(d) level using Gaussian 09.


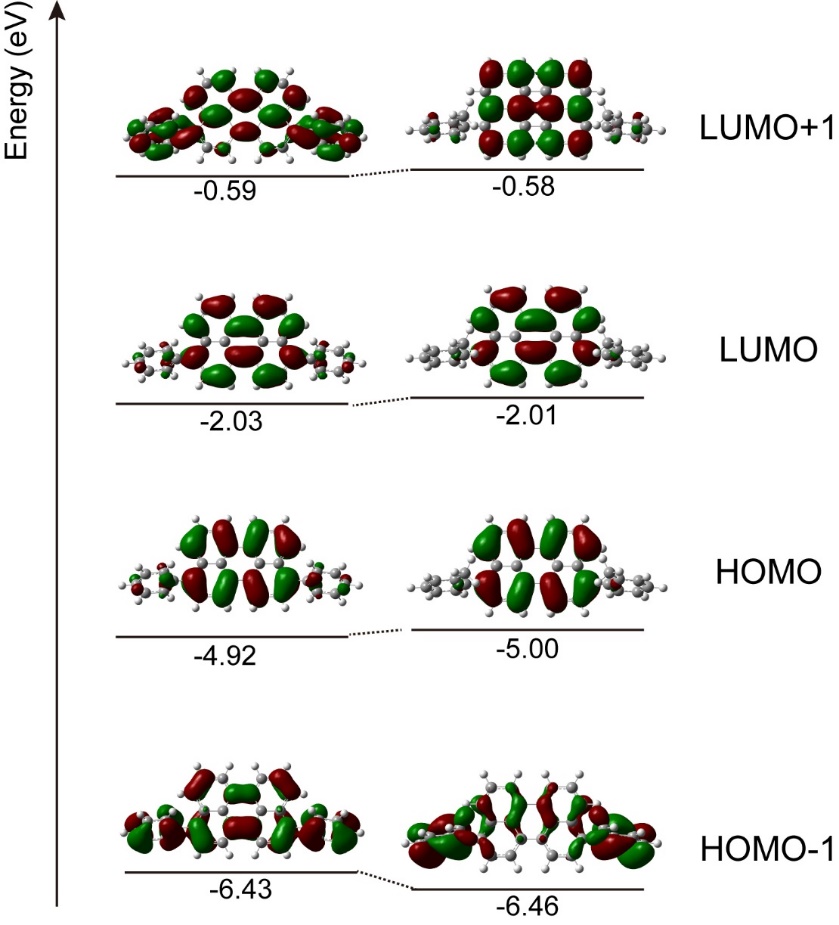


**Supplementary Figure 7**. Frontier molecular orbitals of Py2 (left) and Py4 (right) on the basis of the optimized ground-state geometry at the B3LYP/6-31G(d) level using Gaussian 09.


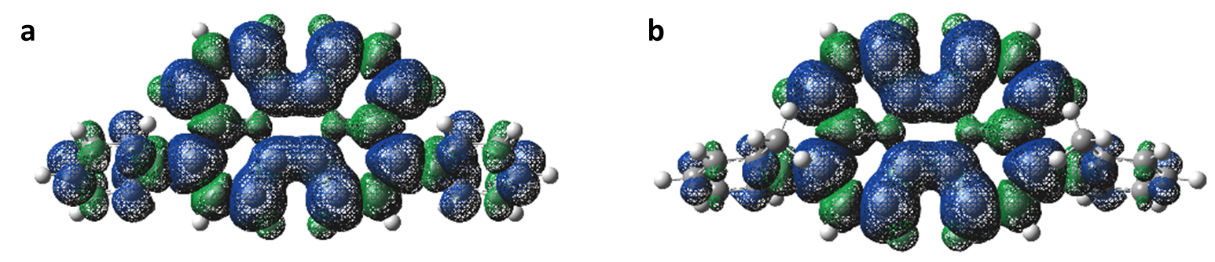


**Supplementary Figure 8**. Isosurfaces of spin density at the optimized triplet state geometries in (a) Py2 and (b) Py4 (CPCM model). Calculated by DFT at B3LYP/6-31G(d) level with Gaussian 09.


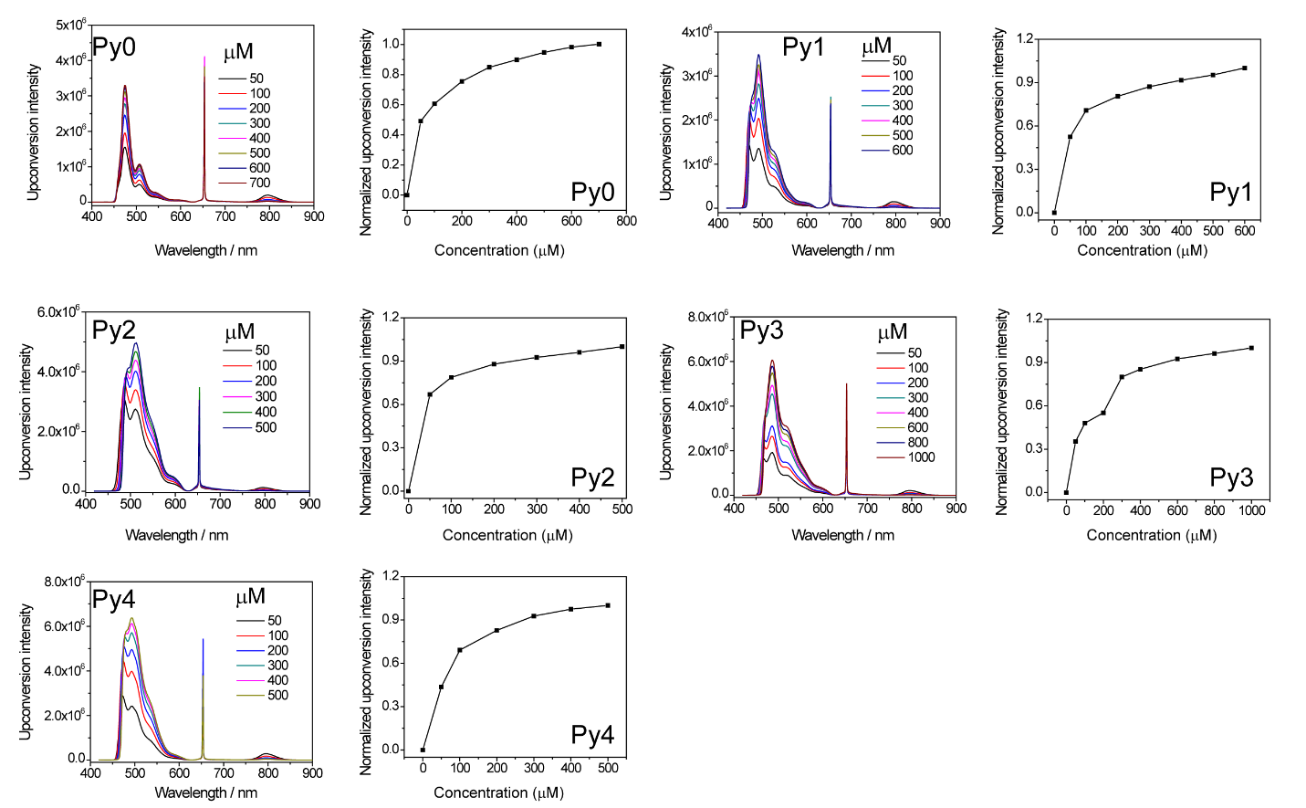


**Supplementary Figure 9**. The upconversion emission spectra of PdTPBP (10 µM) with different concentrations of Py0-Py4 in degassed toluene and the related quantitative analysis for the relationship between upconversion intensity and concentration of annihilator.


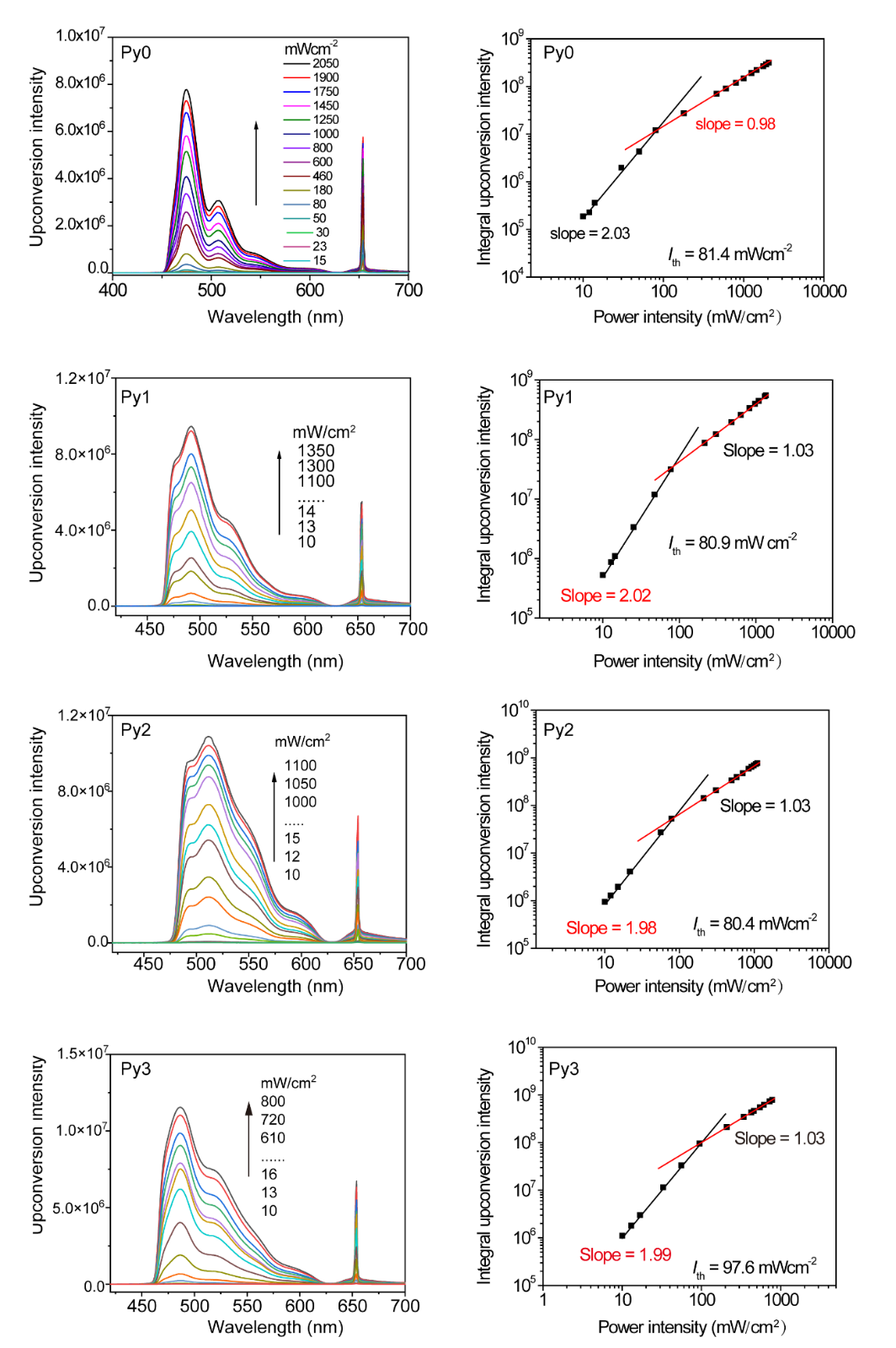


**Supplementary Figure 10**. Incident light power dependence study of TTA-upconversion for PdTPBP (10 μM) with Py0-Py3 at optimized concentration in degassed toluene, respectively. Double-logarithmic plot of integrated upconversion emission intensity as a function of 653 nm excitation power density was drawn. Solid lines illustrate a slope about 2 (black, quadratic) and a slope about 1 (red, linear).


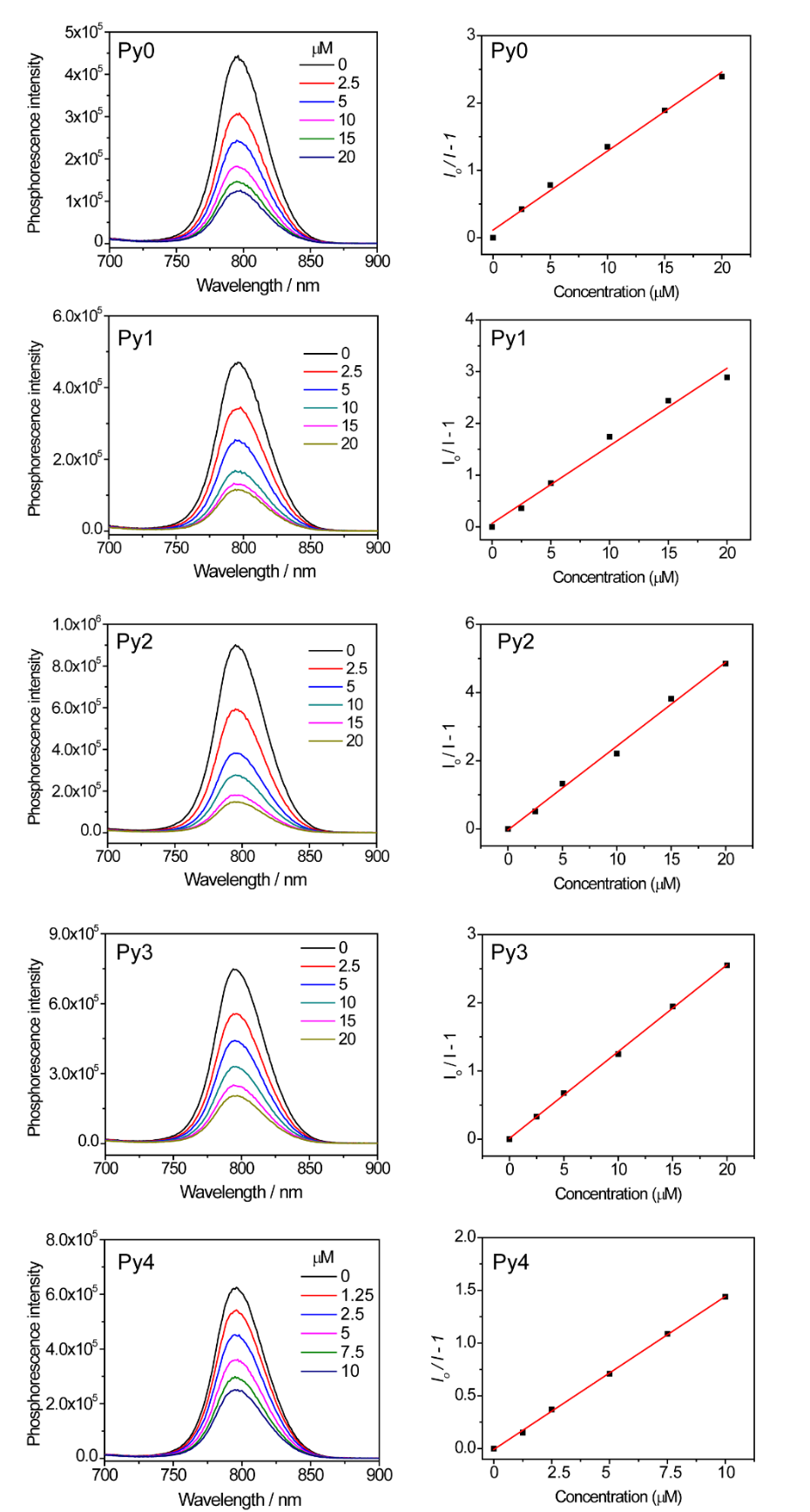


**Supplementary Figure 11**. Phosphorescence intensity quenching of PdTPBP via titration of annihilators (Py0-Py4) in toluene and the obtained Stern−Volmer plots, *c* (PdTPBP) = 10 µM, *λ*_ex_ = 630 nm.


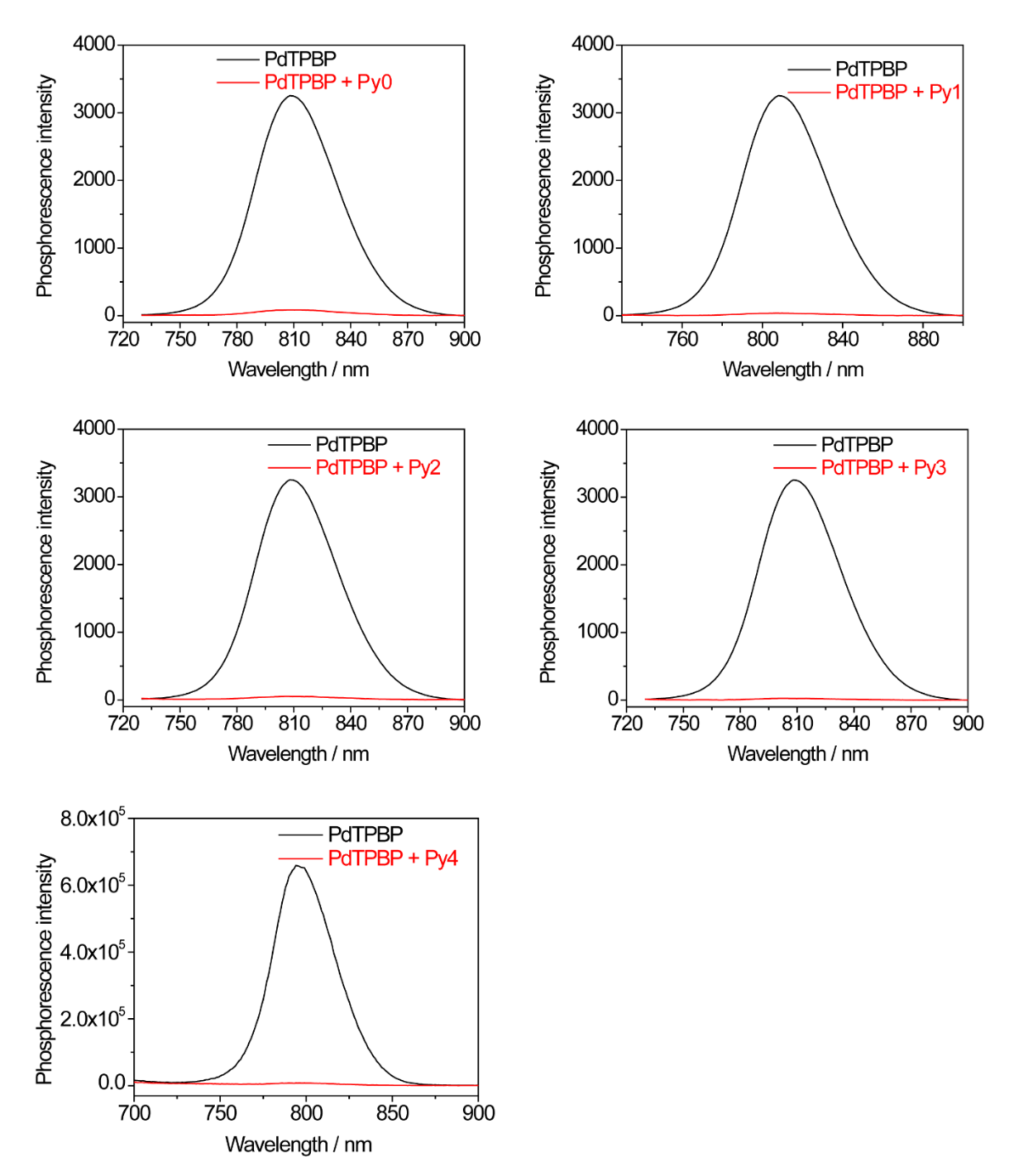


**Supplementary Figure 12**. Phosphorescence emission spectra of PdTPBP without annihilators and in the presence of optimized concentration Py0-Py4, *λ*_ex_ =630 nm, in toluene.


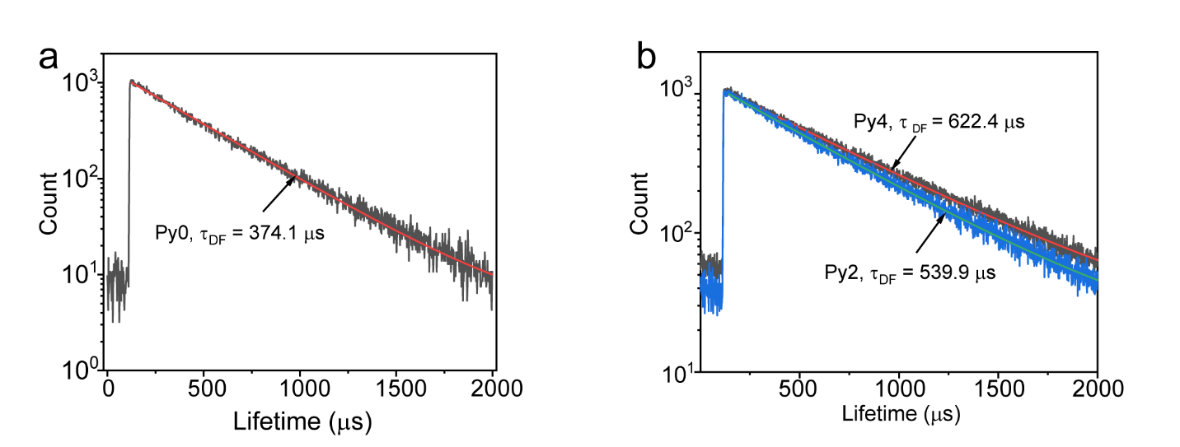


**Supplementary Figure 13**. Upconverted fluorescence lifetime of (a) PdTPBP/Py0 and (b) PdTPBP/Py2, PdTPBP/Py4. A nanosecond pulsed laser 653 nm as excitation source, in argon saturated toluene.


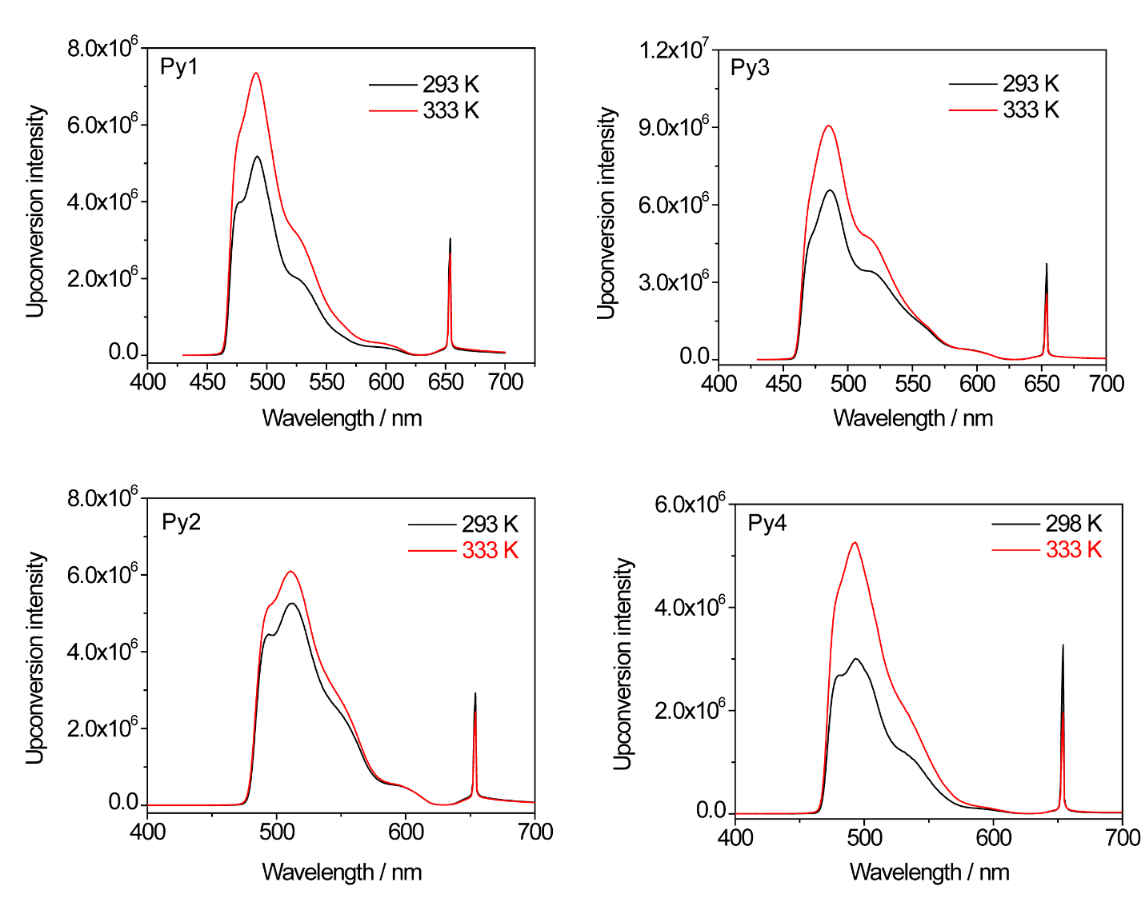


**Supplementary Figure 14**. The temperature effect on TTA-UC spectra of PdTPBP/Py1, PdTPBP/Py2, PdTPBP/Py3, and PdTPBP/Py4 at 293K and 333K.


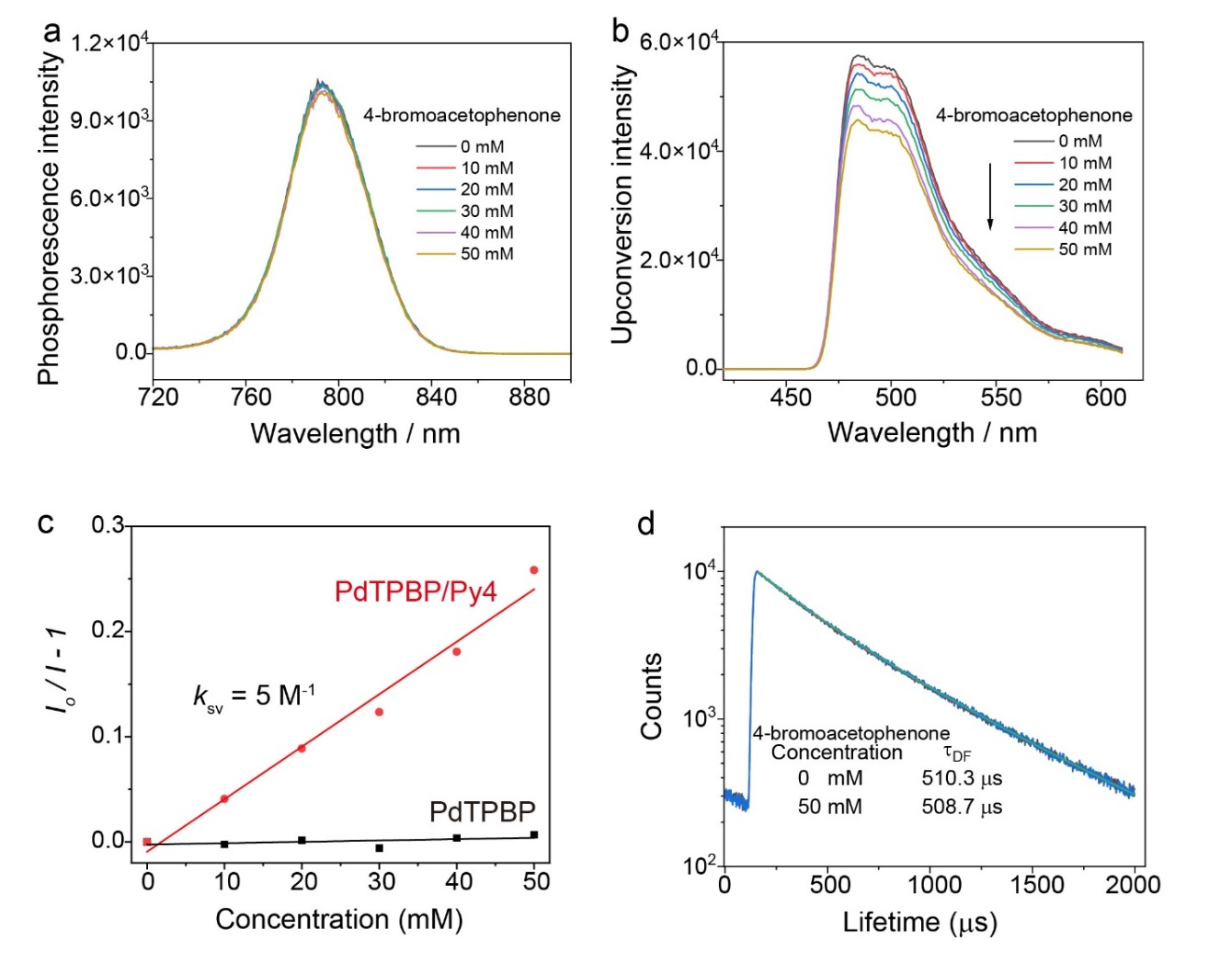


**Supplementary Figure 15**. (a) Phosphorescent emission spectra of PdTPBP in the presence of 4-bromoacetophenone at different concentrations (0. 10, 20, 30, 40, 50 mM); (b) TTA-UC spectra of PdTPBP/Py4 at different concentrations of 4-bromoacetophenone (0. 10, 20, 30, 40, 50 mM); (c) Stern-Volmer quenching plotting of PdTPBP and PdTPBP/Py4 with 4-bromoacetophenone; (d) TTA-UC luminescence lifetime of PdTPBP/Py4 in the absence and presence of 4-bromoacetophenone. 4-bromoacetophenone, 50 mM. Solvent: argon-saturated DMSO, PdTPBP: 10 μM, Py4: 500 μM, *λ*_ex_ = 653 nm.


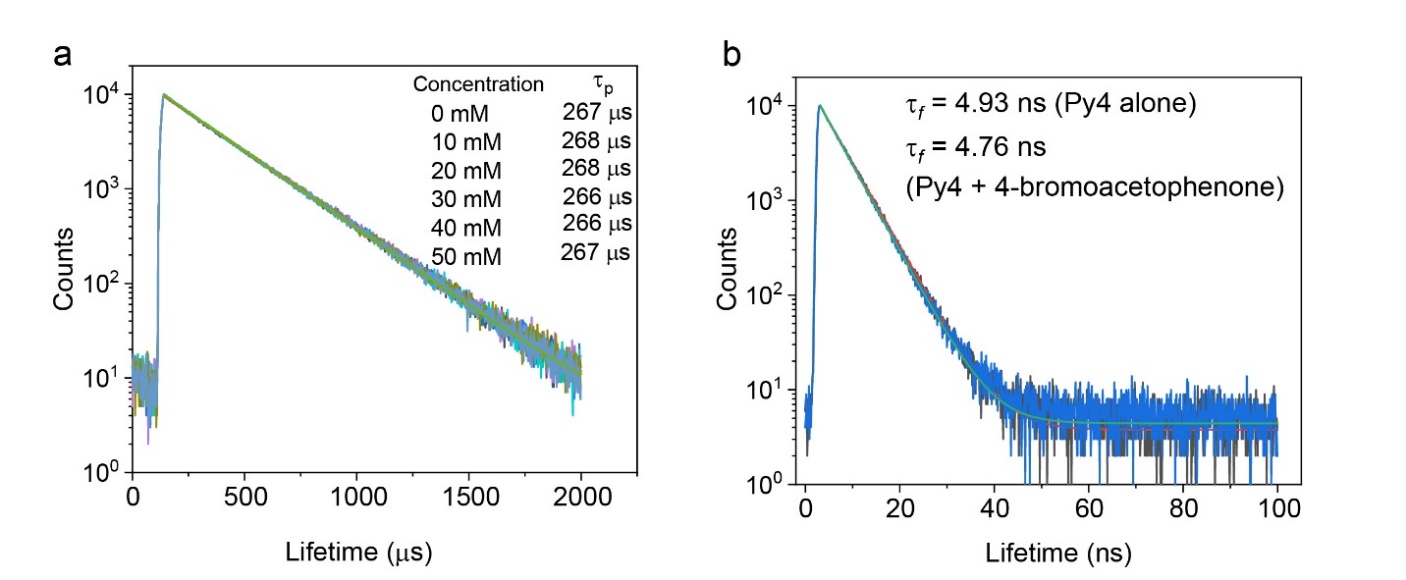


**Supplementary Figure 16**. (a) Phosphorescent lifetime of PdTPBP in the presence of different concentrations of 4-bromoacetophenone, *λ*_ex_ = 653 nm. (b) The fluorescence lifetime of Py4 with or without 4-bromoacetophenone, *λ*_ex_ = 405 nm. Solvent: argon-saturated DMSO, PdTPBP: 10 μM, Py4: 500 μM.

**NMR and ESI-HRMS spectra of photocatalysts and the photocatalyzed products**

**Supplementary Figure 17**. ^1^H NMR of Py1 (500 MHz, CDCl_3_).

**Supplementary Figure 18**. ^13^C NMR of Py1 (125 MHz, CDCl_3_).

**Supplementary Figure 19**. ^1^H NMR of Py2 (500 MHz, CDCl_3_).

**Supplementary Figure 20**. ^13^C NMR of Py2 (125 MHz, CDCl_3_).

**Supplementary Figure 21**. ^1^H NMR of Py3 (500 MHz, CDCl_3_).

**Supplementary Figure 22**. ^13^C NMR of Py3 (125 MHz, CDCl_3_).

**Supplementary Figure 23**. ^1^H NMR of Py4 (500 MHz, CDCl_3_).

**Supplementary Figure 24**. ^13^C NMR of Py4 (125 MHz, CDCl_3_).

**Supplementary Figure 25**. ^1^H NMR of compound **1** (500 MHz, CDCl_3_).

**Supplementary Figure 26**. ^13^C NMR of compound **1** (125 MHz, CDCl_3_)

**Supplementary Figure 27**. ^1^H NMR of compound **2** (500 MHz, CDCl_3_).

**Supplementary Figure 28**. ^13^C NMR of compound **2** (125 MHz, CDCl_3_).

**Supplementary Figure 29**. ^1^H NMR of compound **3** (500 MHz, CDCl_3_).

**Supplementary Figure 30**. ^13^C NMR of compound **3** (125 MHz, CDCl_3_).

**Supplementary Figure 31**. ^1^H NMR of compound **4** (500 MHz, CDCl_3_).

**Supplementary Figure 32**. ^13^C NMR of compound **4** (125 MHz, CDCl_3_).

**Supplementary Figure 33**. ^1^H NMR of compound **5** (500 MHz, CDCl_3_).

**Supplementary Figure 34**. ^13^C NMR of compound **5** (125 MHz, CDCl_3_).

**Supplementary Figure 35**. ^1^H NMR of compound **6** (500 MHz, CDCl_3_).

**Supplementary Figure 36**. ^13^C NMR of compound **6** (125 MHz, CDCl_3_).

**Supplementary Figure 37**. ^1^H NMR of compound **7** (500 MHz, CDCl_3_).

**Supplementary Figure 38**. ^13^C NMR of compound **7** (125 MHz, CDCl_3_).

**Supplementary Figure 39**. ^1^H NMR of compound **8** (500 MHz, CDCl_3_).

**Supplementary Figure 40**. ^13^C NMR of compound **8** (125 MHz, CDCl_3_).

**Supplementary Figure 41**. ^1^H NMR of compound **9** (500 MHz, CDCl_3_).

**Supplementary Figure 42**. ^13^C NMR of compound **9** (125 MHz, CDCl_3_).

**Supplementary Figure 43**. ^1^H NMR of compound **10** (500 MHz, CDCl_3_).

**Supplementary Figure 44**. ^13^C NMR of compound **10** (125 MHz, CDCl_3_).

**Supplementary Figure 45**. ^1^H NMR of compound **11** (500 MHz, CDCl_3_).

**Supplementary Figure 46**. ^13^C NMR of compound **11** (125 MHz, CDCl_3_).

**Supplementary Figure 47**. ^1^H NMR of compound **12** (500 MHz, CDCl_3_).

**Supplementary Figure 48**. ^13^C NMR of compound **12** (125 MHz, CDCl_3_).

**Supplementary Figure 49**. ^1^H NMR of compound **13** (500 MHz, CDCl_3_).

**Supplementary Figure 50**. ^13^C NMR of compound **13** (125 MHz, CDCl_3_).

**Supplementary Figure 51**. ^1^H NMR of compound **14** (500 MHz, CDCl_3_).

**Supplementary Figure 52**. ^13^C NMR of compound **14** (125 MHz, CDCl_3_).

**Supplementary Figure 53**. ^1^H NMR of compound **15** (500 MHz, CDCl_3_).

**Supplementary Figure 54**. ^13^C NMR of compound **15** (125 MHz, CDCl_3_).

**Supplementary Figure 55**. ^1^H NMR of compound **16** (500 MHz, CDCl_3_).

**Supplementary Figure 56**. ^13^C NMR of compound **16** (125 MHz, CDCl_3_).

**Supplementary Figure 57**. ^1^H NMR of compound **17** (500 MHz, CDCl_3_).

**Supplementary Figure 58**. ^13^C NMR of compound **17** (125 MHz, CDCl_3_).

**Supplementary Figure 59**. ^31^P NMR of compound **17**.

**Supplementary Figure 60**. ESI-HRMS of Py1.

**Supplementary Figure 61**. ESI-HRMS of Py2.

**Supplementary Figure 62**. ESI-HRMS of Py3.

**Supplementary Figure 63**. ESI-HRMS of Py4.

**Supplementary Figure 64**. ESI-HRMS of compound **1**.

**Supplementary Figure 65**. ESI-HRMS of compound **2**.

**Supplementary Figure 66**. ESI-HRMS of compound **4**.

**Supplementary Figure 67**. ESI-HRMS of compound **5**.

**Supplementary Figure 68**. ESI-HRMS of compound **6**.

**Supplementary Figure 69**. ESI-HRMS of compound **7**.

**Supplementary Figure 70**. ESI-HRMS of compound **8**.

**Supplementary Figure 71**. ESI-HRMS of compound **9**.

**Supplementary Figure 72**. ESI-HRMS of compound **10**.

**Supplementary Figure 73**. ESI-HRMS of compound **11**.

**Supplementary Figure 74**. ESI-HRMS of compound **13**.

**Supplementary Figure 75**. ESI-HRMS of compound **14**.

**Supplementary Figure 76**. ESI-HRMS of compound **15**.

**Supplementary Figure 77**. ESI-HRMS of compound **16**.

**Supplementary Figure 78**. ESI-HRMS of compound **17**.

# Supplementary References

1 Cui, X. *et al.* Perylene-Derived Triplet Acceptors with Optimized Excited State Energy Levels for Triplet–Triplet Annihilation Assisted Upconversion. *J. Org. Chem.* **79**, 2038-2048 (2014).

2 Singh, V. K. *et al.* Highly efficient organic photocatalysts discovered via a computer-aided-design strategy for visible-light-driven atom transfer radical polymerization. *Nat. Catal.* **1**, 794-804 (2018).

3 Awwad, N., Bui, A. T., Danilov, E. O. & Castellano, F. N. Visible-Light-Initiated Free-Radical Polymerization by Homomolecular Triplet-Triplet Annihilation. *Chem* **6**, 3071-3085 (2020).

4 Liu, Q. *et al.* A General Strategy for Biocompatible, High-Effective Upconversion Nanocapsules Based on Triplet–Triplet Annihilation. *J. Am. Chem. Soc.* **135**, 5029-5037 (2013).

5 Singh-Rachford, T. N. & Castellano, F. N. Photon upconversion based on sensitized triplet–triplet annihilation. *Coord. Chem. Rev.* **254**, 2560-2573 (2010).

6 Deng, F., Sun, W. & Castellano, F. N. Texaphyrin sensitized near-IR-to-visible photon upconversion. *Photochem. Photobiol. Sci.* **13**, 813-819 (2014).

7 Cui, X., Zhao, J., Yang, P. & Sun, J. Zinc(ii) tetraphenyltetrabenzoporphyrin complex as triplet photosensitizer for triplet–triplet annihilation upconversion. *Chem.Comm.* **49**, 10221-10223 (2013).

8 Mattiello, S. *et al.* Self-Assembled Dual Dye-Doped Nanosized Micelles for High-Contrast Up-Conversion Bioimaging. *Adv. Funct. Mater.* **26**, 8447-8454 (2016).

9 Alves, J., Feng, J., Nienhaus, L. & Schmidt, T. W. Challenges, progress and prospects in solid state triplet fusion upconversion. *J. Mater. Chem. C* **10**, 7783-7798 (2022).

10 López-Calixto, C. G., Liras, M., de la Peña O’Shea, V. A. & Pérez-Ruiz, R. Synchronized biphotonic process triggering CC coupling catalytic reactions. *Appl. Catal. B* **237**, 18-23 (2018).
