# Supplementary material for: Red light-driven electron sacrificial agents-free photoreduction of inert aryl halides via triplet-triplet annihilation
Source: Nat Commun. 2023 Feb 27;14:1102. doi: 10.1038/s41467-023-36679-7 (PMC9968713; doi:10.1038/s41467-023-36679-7)
Supplement: Supplementary file 3 — Description of Additional Supplementary Files [file 41467_2023_36679_MOESM3_ESM.pdf]

Description of Additional Supplementary Files

Supplementary Data 1: contains all the coordinates from the theoretical calculations.
