# Supplementary material for: Red light-driven electron sacrificial agents-free photoreduction of inert aryl halides via triplet-triplet annihilation
Source: Nat Commun. 2023 Feb 27;14:1102. doi: 10.1038/s41467-023-36679-7 (PMC9968713; doi:10.1038/s41467-023-36679-7)
Supplement: Supplementary file 4 — Supplementary Data 1 [file 41467_2023_36679_MOESM4_ESM.docx]

**Py1** Symbolic Z-matrix:

Charge = 0 Multiplicity = 1

C -0.84251 2.9444 -0.30044

C 0.53929 2.70893 -0.23134

C 1.05604 1.42339 -0.11076

C 0.14498 0.31606 -0.05752

C -1.27343 0.56274 -0.0868

C -1.73332 1.89822 -0.22822

C 0.63328 -1.02973 0.00702

C -0.29486 -2.06376 0.03263

C 4.00081 -2.80427 0.12861

C -2.19071 -0.5386 -0.01444

C 2.51083 1.17453 -0.04564

C 2.99116 -0.17299 0.01709

C 2.08647 -1.28287 0.0372

C 4.40477 -0.41645 0.06314

C 4.88412 -1.74966 0.11708

C -1.67537 -1.82259 0.02556

C 2.61466 -2.56963 0.09104

C 3.44077 2.21021 -0.04079

C 4.82547 1.96819 0.00858

C 5.30418 0.6795 0.05593

C -3.66867 -0.35727 0.02491

C -4.28863 0.42115 1.01792

C -5.6774 0.53319 1.07523

C -6.47639 -0.12992 0.14151

C -5.87507 -0.9091 -0.84804

C -4.48584 -1.02266 -0.90388

H -1.20539 3.96194 -0.41794

H 1.20441 3.56264 -0.29147

H -2.79827 2.08546 -0.29783

H 0.03448 -3.09547 0.07686

H 4.36605 -3.82685 0.16966

H 5.9572 -1.92041 0.14963

H -2.35811 -2.66576 0.08376

H 1.95583 -3.43029 0.10686

H 3.10971 3.24185 -0.07161

H 5.51238 2.81018 0.00878

H 6.3726 0.48223 0.09169

H -3.67519 0.92455 1.75992

H -6.13623 1.13373 1.85644

H -7.55853 -0.0411 0.1862

H -6.48733 -1.42726 -1.58158

H -4.02136 -1.62155 -1.68264

**Py2** Symbolic Z-matrix:

Charge = 0 Multiplicity = 1

C -2.87352 2.97121 -0.28901

C -1.4703 2.97233 -0.27104

C -0.73921 1.79152 -0.19072

C -1.44961 0.54853 -0.11104

C -2.88943 0.55039 -0.09184

C -3.57281 1.7893 -0.20337

C -0.73698 -0.69271 -0.06521

C -1.47365 -1.87087 -0.02166

C 2.87445 -1.86881 0.01131

C -3.6028 -0.69144 -0.00054

C 0.73921 1.79152 -0.1907

C 1.44961 0.54853 -0.11104

C 0.73698 -0.69271 -0.06521

C 2.88943 0.5504 -0.09183

C 3.6028 -0.69144 -0.00055

C -2.87444 -1.86881 0.01132

C 1.47365 -1.87087 -0.02167

C 1.4703 2.97233 -0.27099

C 2.87352 2.97122 -0.28895

C 3.57281 1.7893 -0.20333

C -5.08751 -0.76583 0.08899

C -5.79879 -0.09976 1.10241

C -7.18349 -0.22734 1.20662

C -7.88737 -1.02354 0.30076

C -7.19432 -1.69398 -0.70824

C -5.80901 -1.5671 -0.8115

C 5.08751 -0.76583 0.08898

C 5.80901 -1.56707 -0.81152

C 7.19432 -1.69396 -0.70827

C 7.88737 -1.02354 0.30075

C 7.18349 -0.22737 1.20663

C 5.79879 -0.09979 1.10242

H -3.40629 3.91417 -0.37786

H -0.96383 3.92815 -0.33505

H -4.65588 1.79522 -0.23565

H -0.97244 -2.83144 0.00829

H 3.40105 -2.81647 0.08261

H -3.40105 -2.81647 0.08264

H 0.97244 -2.83144 0.00826

H 0.96382 3.92816 -0.33498

H 3.40629 3.91417 -0.37778

H 4.65588 1.79523 -0.2356

H -5.25671 0.50532 1.82366

H -7.71232 0.2901 2.0029

H -8.96659 -1.12149 0.38198

H -7.73255 -2.31399 -1.42051

H -5.27525 -2.08105 -1.60638

H 5.27525 -2.08101 -1.60641

H 7.73255 -2.31395 -1.42056

H 8.96659 -1.1215 0.38196

H 7.71232 0.29006 2.00292

H 5.2567 0.50528 1.82368

**Py3** Symbolic Z-matrix:

Charge = 0 Multiplicity = 1

C -0.72746 2.9103 -0.28298

C 0.6585 2.69273 -0.22087

C 1.19624 1.41222 -0.14168

C 0.30211 0.29066 -0.11647

C -1.11723 0.51714 -0.16598

C -1.60146 1.84781 -0.25907

C 0.80534 -1.04975 -0.05308

C -0.10959 -2.09553 -0.05929

C 4.19395 -2.7765 0.14927

C -2.02053 -0.59403 -0.14146

C 2.65446 1.18118 -0.08839

C 3.15114 -0.16017 -0.01205

C 2.2613 -1.28217 0.01048

C 4.56749 -0.38505 0.04429

C 5.06354 -1.7109 0.12372

C -1.49466 -1.8717 -0.10307

C 2.8053 -2.56051 0.09405

C 3.57214 2.22744 -0.10731

C 4.95967 2.00311 -0.05414

C 5.45353 0.72151 0.02073

C -3.50539 -0.41101 -0.1829

C -4.23687 -0.00482 0.95668

C -5.62835 0.11237 0.85078

C -6.29854 -0.16359 -0.34143

C -5.5757 -0.57239 -1.46054

C -4.18884 -0.695 -1.37391

C -3.55192 0.27997 2.27417

H -1.10485 3.92675 -0.35574

H 1.30974 3.55891 -0.24343

H -2.67166 2.01338 -0.32228

H 0.23264 -3.12359 -0.02668

H 4.57195 -3.79328 0.21271

H 6.13853 -1.86713 0.16526

H -2.16926 -2.7236 -0.0955

H 2.15699 -3.42893 0.12063

H 3.22908 3.25404 -0.16354

H 5.63652 2.85302 -0.07202

H 6.52414 0.53784 0.06319

H -6.1964 0.41882 1.72637

H -7.37976 -0.06423 -0.39104

H -6.08393 -0.79519 -2.3949

H -3.61656 -1.01062 -2.24239

H -2.91433 -0.55626 2.58304

H -2.90464 1.16308 2.21378

H -4.28722 0.45704 3.06519

**Py4**  Symbolic Z-matrix:

Charge = 0 Multiplicity = 1

C -2.87422 2.96318 -0.13381

C -1.4717 2.96434 -0.06272

C -0.73842 1.78237 -0.02977

C -1.44571 0.53555 -0.06039

C -2.88236 0.53632 -0.12166

C -3.57019 1.77661 -0.16627

C -0.73633 -0.70851 -0.0403

C -1.47264 -1.8855 -0.10362

C 2.87518 -1.88254 0.16077

C -3.59763 -0.70397 -0.15556

C 0.7384 1.78238 0.02996

C 1.44571 0.53556 0.0605

C 0.73634 -0.70851 0.04036

C 2.88236 0.53635 0.12176

C 3.59765 -0.70395 0.15555

C -2.87516 -1.88257 -0.16079

C 1.47266 -1.88549 0.10364

C 1.47167 2.96436 0.06299

C 2.87418 2.96321 0.1341

C 3.57016 1.77664 0.16648

C -5.09218 -0.75629 -0.21393

C -5.89031 -0.5143 0.9277

C -7.28173 -0.61289 0.8033

C -7.88759 -0.94385 -0.40919

C -7.09774 -1.19074 -1.53049

C -5.70988 -1.09746 -1.42567

C 5.0922 -0.75629 0.21384

C 5.89028 -0.51404 -0.92778

C 7.2817 -0.61274 -0.80349

C 7.88762 -0.94403 0.40889

C 7.09782 -1.19117 1.53016

C 5.70996 -1.0978 1.42544

C -5.27204 -0.17636 2.26531

C 5.27197 -0.17576 -2.26529

H -3.40658 3.90988 -0.16895

H -0.96687 3.92306 -0.04024

H -4.65277 1.77467 -0.23606

H -0.97143 -2.84668 -0.10851

H 3.40533 -2.83036 0.19896

H -3.4053 -2.83039 -0.19904

H 0.97146 -2.84668 0.10849

H 0.96682 3.92307 0.04058

H 3.40653 3.90991 0.16932

H 4.65274 1.77471 0.2363

H -7.90018 -0.43403 1.6801

H -8.9704 -1.01321 -0.47297

H -7.55478 -1.45385 -2.48068

H -5.08594 -1.28535 -2.29559

H 7.90011 -0.43369 -1.68027

H 8.97043 -1.01345 0.47259

H 7.55489 -1.45455 2.48026

H 5.08607 -1.28587 2.29536

H -4.50355 -0.9052 2.54681

H -4.78382 0.80545 2.25109

H -6.03156 -0.16125 3.05312

H 4.78381 0.80608 -2.25082

H 6.03146 -0.16051 -3.05313

H 4.50342 -0.90448 -2.54693
